# Greater white matter degeneration and lower structural connectivity in non-amnestic vs. amnestic Alzheimer’s disease

**DOI:** 10.3389/fnins.2024.1353306

**Published:** 2024-03-18

**Authors:** Jeffrey S. Phillips, Nagesh Adluru, Moo K. Chung, Hamsanandini Radhakrishnan, Christopher A. Olm, Philip A. Cook, James C. Gee, Katheryn A. Q. Cousins, Sanaz Arezoumandan, David A. Wolk, Corey T. McMillan, Murray Grossman, David J. Irwin

**Affiliations:** ^1^Penn Frontotemporal Degeneration Center, Perelman School of Medicine, University of Pennsylvania, Philadelphia, PA, United States; ^2^Department of Neurology, Perelman School of Medicine, University of Pennsylvania, Philadelphia, PA, United States; ^3^Waisman Center, University of Wisconsin-Madison, Madison, WI, United States; ^4^Department of Radiology, University of Wisconsin-Madison, Madison, WI, United States; ^5^Department of Biostatistics and Medical Informatics, School of Medicine and Public Health, University of Wisconsin-Madison, Madison, WI, United States; ^6^Penn Image Computing and Science Laboratory, Department of Radiology, Perelman School of Medicine, University of Pennsylvania, Philadelphia, PA, United States; ^7^Penn Memory Center, University of Pennsylvania Health System, Philadelphia, PA, United States

**Keywords:** diffusion MRI (dMRI), non-amnestic Alzheimer’s disease, logopenic variant of primary progressive aphasia (lvPPA), posterior cortical atrophy (PCA), corticobasal syndrome (CBS), behavioral variant Alzheimer’s disease, network topology analysis

## Abstract

**Introduction:**

Multimodal evidence indicates Alzheimer’s disease (AD) is characterized by early white matter (WM) changes that precede overt cognitive impairment. WM changes have overwhelmingly been investigated in typical, amnestic mild cognitive impairment and AD; fewer studies have addressed WM change in atypical, non-amnestic syndromes. We hypothesized each non-amnestic AD syndrome would exhibit WM differences from amnestic and other non-amnestic syndromes.

**Materials and methods:**

Participants included 45 cognitively normal (CN) individuals; 41 amnestic AD patients; and 67 patients with non-amnestic AD syndromes including logopenic-variant primary progressive aphasia (lvPPA, *n* = 32), posterior cortical atrophy (PCA, *n* = 17), behavioral variant AD (bvAD, *n* = 10), and corticobasal syndrome (CBS, *n* = 8). All had T1-weighted MRI and 30-direction diffusion-weighted imaging (DWI). We performed whole-brain deterministic tractography between 148 cortical and subcortical regions; connection strength was quantified by tractwise mean generalized fractional anisotropy. Regression models assessed effects of group and phenotype as well as associations with grey matter volume. Topological analyses assessed differences in persistent homology (numbers of graph components and cycles). Additionally, we tested associations of topological metrics with global cognition, disease duration, and DWI microstructural metrics.

**Results:**

Both amnestic and non-amnestic patients exhibited lower WM connection strength than CN participants in corpus callosum, cingulum, and inferior and superior longitudinal fasciculi. Overall, non-amnestic patients had more WM disease than amnestic patients. LvPPA patients had left-lateralized WM degeneration; PCA patients had reductions in connections to bilateral posterior parietal, occipital, and temporal areas. Topological analysis showed the non-amnestic but not the amnestic group had more connected components than controls, indicating persistently lower connectivity. Longer disease duration and cognitive impairment were associated with more connected components and fewer cycles in individuals’ brain graphs.

**Discussion:**

We have previously reported syndromic differences in GM degeneration and tau accumulation between AD syndromes; here we find corresponding differences in WM tracts connecting syndrome-specific epicenters. Determining the reasons for selective WM degeneration in non-amnestic AD is a research priority that will require integration of knowledge from neuroimaging, biomarker, autopsy, and functional genetic studies. Furthermore, longitudinal studies to determine the chronology of WM vs. GM degeneration will be key to assessing evidence for WM-mediated tau spread.

## Introduction

Alzheimer’s disease (AD) has classically been interpreted as a disease of the grey matter (GM). However, early postmortem studies of AD (Kowall and Kosik, 1987) noted significant changes in the white matter (WM), and human imaging studies have consistently reported alterations in WM diffusivity and microstructure. WM changes in AD have often been dismissed as consequences of independent but co-occurring microvascular disease (Brun and Englund, 1986). However, neuroimaging investigations of WM change have provided additional perspective on the timecourse of WM changes and their associations with AD biomarkers, indicating that WM changes at least partially reflect demyelination (Fornari et al., 2012) or axonal loss (Scheltens et al., 1995; McAleese et al., 2017; Strain et al., 2018; Desmarais et al., 2021) as a result of AD pathologic change. Diffusion weighted MRI (DWI) studies have shown that WM changes in amnestic AD correspond to the topography of GM disease (Pichet Binette et al., 2021; Wen et al., 2021). Furthermore, positron emission tomography (PET) imaging using ligands for amyloid- β and tau have shown that WM changes are statistically associated with GM accumulation of the two hallmark proteins in AD (Jacobs et al., 2018; Shigemoto et al., 2018; Pichet Binette et al., 2021).

In typical, amnestic AD, WM changes have demonstrated utility as both diagnostic and prognostic features. Distinct patterns of WM degeneration discriminate clinically similar patients who are positive vs. negative for cerebrospinal fluid markers of AD (McMillan et al., 2012). Decreases in the integrity of the cingulum, fornix, and precuneus and parahippocampal WM have predicted future conversion from normal cognition to mild cognitive impairment (MCI) (Zhuang et al., 2012). WM hyperintensities—which typically reflect ischemic vascular disease in cognitively normal individuals, but are associated with AD pathophysiology in people with clinically-diagnosed amnestic AD (McAleese et al., 2017)—predicted 1-year cognitive decline in the Alzheimer’s Disease Neuroimaging Initiative dataset (Carmichael et al., 2010).

One lens for interpreting WM changes in AD is the hypothesis of prion-like tau spread, which posits a key role of the WM as the avenue for interneuronal spread of tau pathology (Ahmed et al., 2014). Tau may spread intra-axonally, diffuse through the interstitial fluid, or be spread by glial cells (Guo and Lee, 2014; Mudher et al., 2017; Amro et al., 2021). In the case of intra-axonal propagation, seed-competent soluble tau may interact with healthy tau to produce creeping spread of toxic proteins along the length of axons, destabilizing microtubules in the process and affecting both axonal transport and the structural integrity of the axon (Ozcelik et al., 2016; Mudher et al., 2017). Axonal degeneration, in turn, could affect multiple DWI metrics: for example, it may produce reductions in neurite density metrics (Colgan et al., 2016), fiber dispersion (Veale et al., 2021), fractional anisotropy, and mean or radial diffusivity (Wells et al., 2015; Wen et al., 2021). Transneuronal spread of tau is supported by *in vitro* studies demonstrating that tau antibodies prevent the spread of tau pathology (Mudher et al., 2017; Nobuhara et al., 2017).

We thus posit that the spread of tau along WM tracts connecting one region to another is likely be associated with degeneration of the tract. However, investigations of WM changes in typical, amnestic AD involve a limited set of anatomical hypotheses: canonical Braak staging forms the expected topography of GM disease spread, and by extension the WM projections that are most likely to exhibit disease-related changes. Individualized approaches to disease progression modeling (Franzmeier et al., 2020) mitigate this limitation by acknowledging person-to-person variability in disease anatomy, though study selection criteria may still bias results to representing typical, late-onset AD anatomy.

Rare non-amnestic AD syndromes with focal cortical disease represent an opportunity for repeated tests of the axonal spread hypothesis with syndrome-specific GM regions and WM tracts of interest. However, WM change in non-amnestic AD is understudied. Patterns of GM atrophy and tau accumulation are known to differ between non-amnestic AD clinical syndromes and amnestic AD (Ossenkoppele et al., 2015a; Phillips et al., 2018a), but there is less evidence regarding whether patterns of WM degeneration exhibit corresponding phenotypic differences, and whether those WM degeneration patterns reflect the topography of GM disease in each clinical syndrome. Early-onset amnestic AD cases—who are more likely to have atypical, non-amnestic clinical presentations (Balasa et al., 2011; Mendez et al., 2012)—have larger reductions in WM integrity on diffusion MRI than late-onset cases, suggesting an underappreciated role for WM in atypical AD (Sirkis et al., 2022). Previous studies of WM degeneration in early-onset and atypical AD include Caso et al. (2015), who found that non-amnestic AD patients had WM degeneration out of proportion with their GM atrophy; however, they did not find differences in WM degeneration between non-amnestic AD syndromes. Additionally, Singh et al. (2023) reported that patients with logopenic-variant primary progressive aphasia (lvPPA) and posterior cortical atrophy (PCA) had both shared and syndrome-specific patterns of WM degeneration relative to cognitively unimpaired participants; however, this study did not assess differences relative to amnestic AD. Finally, Gatto et al. (2022) reported increased mean diffusivity of right superior longitudinal fasciculus among lvPPA patients relative to healthy controls and individuals with progressive supranuclear palsy, demonstrating both the pathological and anatomical specificity of diffusion MRI measures.

**Figure 1:**
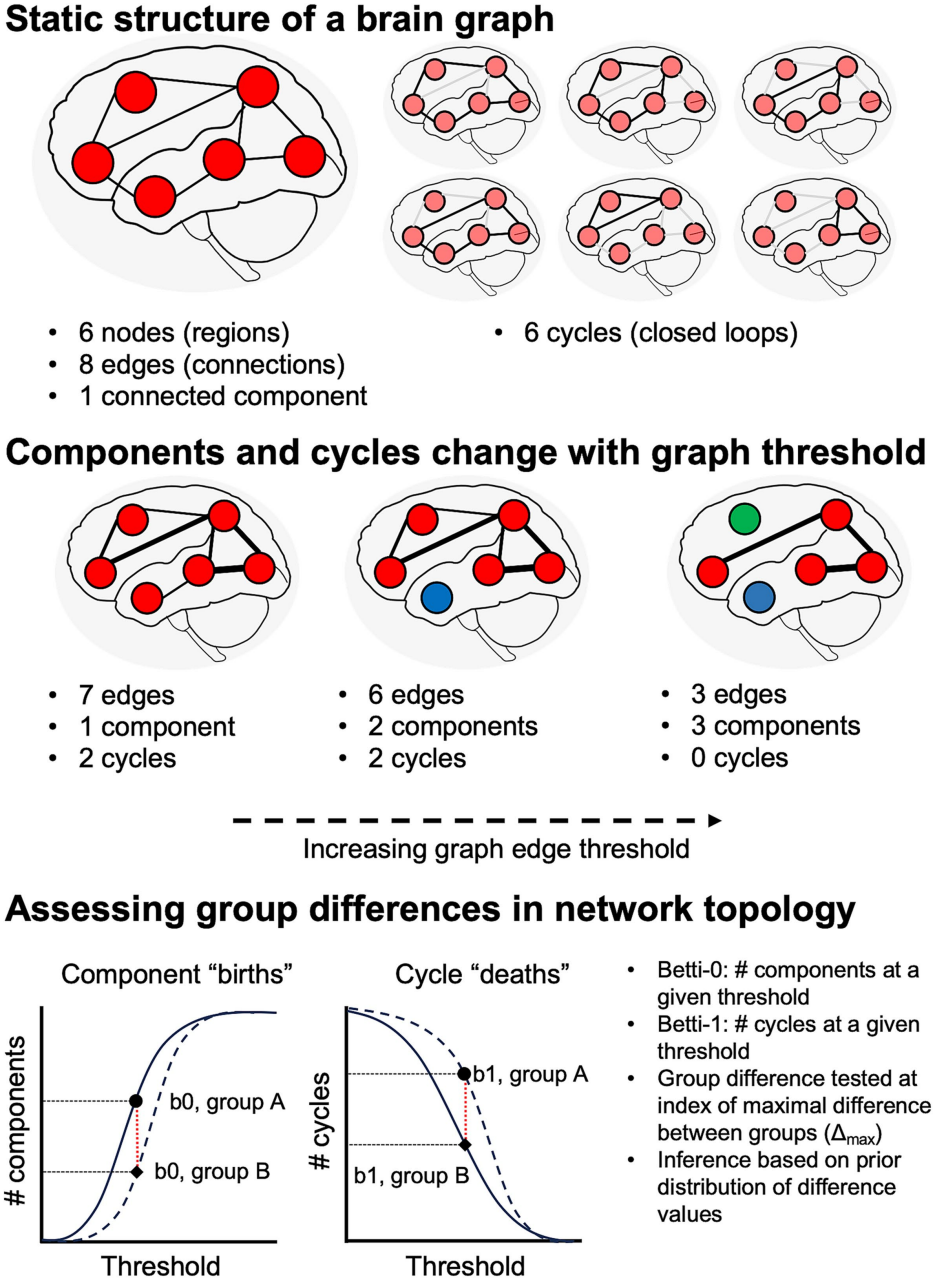
Schematic illustration of network topology analysis. Top: at a given graph threshold, we can quantify the number of nodes, edges, distinct graph components, and cycles (i.e., closed loops). If there is a path from each node to all other nodes, the graph has a single connected component. Middle: analysis of persistent homology requires assessing graph features over a range of thresholds. Increasing graph thresholds cause fragmentation of the graph into more components (differentiated from each other by red, blue, and green colors). Bottom: numbers of components and cycles at a given threshold are captured by Betti-0 and Betti-1 numbers, respectively.

The current study thus aimed to test two hypotheses derived from transneuronal disease spread models: (1) that WM degeneration patterns differ in syndrome-specific fashion; and (2) that these WM changes are quantitatively associated with GM atrophy. Additionally, we aimed to assess global differences in network topology between the cognitively normal, amnestic AD, and non-amnestic AD groups. In contrast to studies that have used voxelwise or regional averages of diffusion microstructure metrics, we adopted a connectomic approach that allowed us to localize between-group differences to WM fiber tracts connecting specific regions. This connectomic approach also allowed us to perform topological analyses on participants’ brain graphs and contrast persistent homology between groups. Persistent homology—i.e., the study of similar shapes and structures that persist over a range of scales—is an algebraic topology method for extracting geometrically invariant signals from brain networks (Chung et al., 2017). It captures the evolution of connectivity structures (such as connected components, cliques, loops, and cavities) as edges are progressively removed from the graph, from the smallest to largest edge weight (a process formally known as graph filtration), thereby revealing connectivity features that persist over many sub-networks (Figure 1). The two main features typically formulated in persistent homology of brain networks are the number of connected components, also formally known as Betti-0 numbers, and the number of loops (cycles), formally known as Betti-1 numbers. Connected components and loops as well as more complex structures like cliques form an important basis for a network’s functionality and efficiency in propagating information or even disease-causing agents (Sizemore et al., 2018).

Based on our prior studies of GM atrophy and tau accumulation in non-amnestic AD, as well as relevant prior work (Caso et al., 2015; Singh et al., 2023), we hypothesized syndrome-specific patterns of WM degeneration in non-amnestic AD. In amnestic AD, we hypothesized that patients would have degeneration of WM projections from limbic and default-mode network regions, particularly the parahippocampal cingulum (Pichet Binette et al., 2021; Raghavan et al., 2022). In lvPPA, we predicted patients would have left-lateralized WM degeneration affecting projections from posterior temporal and inferior parietal cortex, an established epicenter of disease in this form of aphasia (Conca et al., 2022; Mandelli et al., 2023). In contrast, we predicted that PCA patients would exhibit a posterior-to-anterior gradient of bilateral WM degeneration, reflecting early degeneration of occipital and parietal areas along with their WM projections (Migliaccio et al., 2012, 2019; Phillips et al., 2019). Corticobasal syndrome (CBS) due to AD displays a similar distribution of GM disease as in PCA (Phillips et al., 2018a); we thus again anticipated degeneration of projections from parietal and posterior temporal cortex. In behavioral variant AD (bvAD), we and others have found both prefrontal and tempoparietal GM atrophy (Ossenkoppele et al., 2015b; Phillips et al., 2018a; Dominguez Perez et al., 2022); accordingly, we predicted that bvAD would be characterized by degeneration of projections from bilateral dorsolateral prefrontal and orbitofrontal cortex; middle and superior temporal gyri; and medial parietal cortex.

## Materials and methods

### Participants

Participants were retrospectively selected for availability of T1-weighted MRI and DWI as well as a relevant clinical diagnosis. Cognitively impaired patients were recruited from the Cognitive Neurology Clinic at the University of Pennsylvania’s Perelman School of Medicine; cognitively normal (CN) participants were recruited from among patients’ families and from the surrounding community. All participants and/or caregivers gave informed consent to research in accordance with the standards of the University of Pennsylvania Institutional Review Board. CN participants (*n* = 45) were required to have a Mini-Mental Status Exam (MMSE) total score > = 27 or normal cognitive function per clinician judgment. Patients were required to have a clinical diagnosis of amnestic mild cognitive impairment (aMCI) or amnestic AD (collectively, amnestic AD; *n* = 41); or a non-amnestic AD syndrome, including lvPPA (*n* = 32), PCA (*n* = 17), CBS (*n* = 8), or bvAD (*n* = 10). All patients were diagnosed through consensus by expert clinicians from the Penn Frontotemporal Degeneration Center (FTDC) and Alzheimer’s Disease Research Center (ADRC) per published diagnostic criteria (Gorno-Tempini et al., 2011; Lee et al., 2011; McKhann et al., 2011; Rascovsky et al., 2011; Armstrong et al., 2013; Crutch et al., 2017). Evidence of AD neuropathologic change was available for 64 of 67 non-amnestic AD and 30 of 41 amnestic AD patients. Sources of evidence included an amyloid- β concentration < 192 pg/mL in cerebrospinal fluid (amnestic AD, *n* = 19; non-amnestic AD, *n* = 34) using INNOTEST reagent kits and enzyme-linked immunosorbent assay or the INNO-BIA AlzBio3 kit on the xMAP Luminex platform (Shaw et al., 2009; Irwin et al., 2012); an amyloid- β 42/40 ratio < 0.065 (amnestic AD, *n* = 6; non-amnestic AD, *n* = 19), using the Fujirebio Lumipulse immunoassay (Leitão et al., 2019); an amyloid PET scan read as positive by an expert reader (non-amnestic AD, *n* = 1); or a primary postmortem diagnosis of intermediate–high AD neuropathologic change (Montine et al., 2012) (amnestic AD, *n* = 5; non-amnestic AD, *n* = 10). Data from 3 lvPPA and 11 amnestic AD cases without available biomarkers were included based on the strong association of these clinical phenotypes with AD neuropathologic change. In addition to analyses of the full *n* = 153 dataset, we performed subset analyses omitting participants without biomarker results (see *Subset analyses* and Supplementary Tables 5–7). *APOE* genotype was available for 12 controls, 30 amnestic AD cases, and 62 individuals with non-amnestic AD syndromes. Analyses of MRI-based atrophy (Phillips et al., 2018a, 2019) and AV-1451 tau PET (Phillips et al., 2018b, 2021) have been previously published for a subset of participants.

### Neuroimaging data acquisition

MRI data were acquired between May 2007 and April 2017 on a Siemens 3-Tesla TIM Trio scanner at the Hospital of the University of Pennsylvania. All participants had T1-weighted MRI and diffusion-weighted imaging (DWI). T1 images for 150 participants were acquired axially with 1 mm isotropic voxels, flip angle of 15 degrees, repetition time (TR) of 1.62 s, and an echo time (TE) of either 3.09 ms (*n* = 100) or 3.87 ms (*n* = 50). For the 3 remaining participants (1 lvPPA, 2 bvAD), T1 images were acquired were acquired sagittally with a slice thickness of 1.2 mm, in-plane resolution of 1×1 mm, flip angle of 9 degrees, TR = 2.3 s, and TE = 2.95 ms. For DWI, participants underwent one (*n* = 34), two (*n* = 4), or three (*n* = 115) runs of DWI with 1–5 *b* = 0 images and 30 volumes at *b* = 1,000. DWI repetition times ranged from 5 s to 11.4 s; while echo times ranged from 80 to 100 ms, and voxel sizes were 1.9×1.9×2 mm (*n* = 103), 2.2 mm isotropic (*n* = 48), or 2.5 mm isotropic (*n* = 2).

### Neuroimaging data processing

T1-weighted MRIs were processed using the Advanced Normalization Tools (ANTs) package as previously described (Phillips et al., 2019; Shen et al., 2023). For all images, intracranial volume was computed by using SynthStrip (Hoopes et al., 2022) to remove the skull, then taking the volume of the resulting brain and surrounding cerebrospinal fluid. T1-weighted MRI and *B* = 0 images for all participants’ DWI series were visually inspected by JSP to assess data quality and registration to the T1 space. Because fieldmap data had been inconsistently acquired over time, we used the Synthesized b0 for diffusion DIStortion COrrection (Synb0-DISCO)^1^ package (Schilling et al., 2019, 2020) to infer undistorted DWI maps. These synthetic maps and the original diffusion-weighted images were given as inputs to QSIPrep 0.18.0 (Cieslak et al., 2021), which is based on Nipype 1.8.6 (Gorgolewski et al., 2011; Esteban et al., 2022) (RRID:SCR_002502).

Per the request of the QSIPrep developers, we include the following auto-generated methods description, with appropriate edits: T1 images were corrected for intensity non-uniformity using N4 bias field correction from ANTs version 2.4.3 (Tustison et al., 2010). A T1-weighted reference map was computed after registration of T1 images using antsRegistration. The anatomical reference image was reoriented into AC-PC alignment via a 6 degree-of-freedom transform extracted from a full affine registration to the MNI152NLin2009cAsym template. A full nonlinear registration to the template from AC-PC space was estimated via symmetric nonlinear registration (SyN) using antsRegistration. Brain extraction was performed on the T1 image using SynthStrip (Hoopes et al., 2022), and automated segmentation was performed using SynthSeg (Billot et al., 2023) from FreeSurfer version 7.3.1. Any images with a b-value less than 100 s/mm^2^ were treated as a *b* = 0 image. MP-PCA denoising as implemented in MRtrix3’s dwidenoise (Veraart et al., 2016) was applied with a 5-voxel window. After MP-PCA, Gibbs unringing was performed using MRtrix3’s mrdegibbs (Kellner et al., 2016). Following unringing, the mean intensity of the DWI series was adjusted so all the mean intensity of the *b* = 0 images matched across each separate DWI scanning sequence. B1 field inhomogeneity was corrected using dwibiascorrect from MRtrix3 with the N4 algorithm after corrected images were resampled.

FSL (version 6.0.5.1:57b01774)’s eddy was used for head motion and eddy-current correction (Andersson et al., 2016). Eddy was configured with a q-space smoothing factor of 10, a total of 5 iterations, and 1,000 voxels used to estimate hyperparameters. A quadratic first level model and a linear second level model were used to characterize eddy current-related spatial distortion. Q-space coordinates were forcefully assigned to shells, and eddy attempted to separate field offset from subject movement. Shells were aligned post-eddy. Eddy’s outlier replacement was run (Andersson et al., 2016). Data were grouped by slice, only including values from slices determined to contain at least 250 intracerebral voxels. Groups deviating by more than 4 standard deviations from the prediction had their data replaced with imputed values. Data was collected with reversed phase-encode blips, resulting in pairs of images with distortions going in opposite directions. The synthetic, undistorted *b* = 0 image computed with Synb0-DISCO was paired with *b* = 0 images extracted from the DWI scans. From these pairs the susceptibility-induced off-resonance field was estimated using a method similar to that described by Andersson et al. (2003). The fieldmaps were ultimately incorporated into the eddy current and head motion correction interpolation. Final interpolation was performed using the jacobian method (Andersson and Sotiropoulos, 2016).

Several confounding time-series were calculated based on the preprocessed DWI: framewise displacement (FD) using the implementation in Nipype (Power et al., 2014). The head-motion estimates calculated in the correction step were also placed within the corresponding confounds file. Slicewise cross correlation was also calculated. The DWI time-series were resampled to AC-PC alignment, generating a preprocessed DWI run in AC-PC space with 1.5 mm isotropic voxels. Many internal operations of QSIPrep use Nilearn 0.10.1 (Abraham et al., 2014) (RRID:SCR_001362) and Dipy (Garyfallidis et al., 2014). For more details of the pipeline, see the section corresponding to workflows in QSIPrep’s documentation.

Diffusion orientation distribution functions (ODFs) were reconstructed using generalized q-sampling imaging (GQI) (Yeh et al., 2010) with a ratio of mean diffusion distance of 1.25 in DSI Studio (version 94b9c79).^2^ GQI belongs to a family of diffusion MRI reconstruction techniques that produce a voxelwise orientation distribution function (ODF) ODF-based reconstruction methods such as GQI allow superior resolution of WM tracts in areas of crossing fibers and edema than classical diffusion tensor imaging (Yeh et al., 2010; Jin et al., 2019; Ye et al., 2021). Whole-brain deterministic tractography was performed per published methods (Yeh et al., 2013); for each participant, we generated 5 million streamlines with a maximum length of 250 mm, minimum length of 30 mm, random seeding, a step size of 1 mm, a turning angle of 35°, and an automatically calculated quantitative anisotropy threshold. No smoothing or topology-informed pruning was used. Generalized fractional anisotropy (GFA) was averaged over the length of streamlines as a measure of WM connection strength. Regions of interest were defined by a composite atlas space comprising the 100-label, 7-network cortical parcellation of Schaefer et al. (2018) and Yeo et al. (2011); the CIT-168 subcortical atlas (Pauli et al., 2018), comprising the basal ganglia and midbrain nuclei; the thalamic atlas of Najdenovska et al. (2018), based on Human Connectome Project (HCP) data; and the cerebellar atlas of King et al. (2019). GM volume was similarly quanitified for all regions of interest from ANTs-processed T1 MRI.

As a quality control measure, we quantified the frequency with which atlas regions occurred as “islands” in participants’ DWI connectivity matrices (i.e., regions unconnected to any other regions). Because the bilateral habenular and mammillary nuclei constituted islands in more than 25% of scans, they were excluded, yielding symmetric 148 × 148 matrices for analysis. Because GFA-based connectivity matrices are sparse, and the presence of individual connections varied between participants, we adopted a “50% rule” to exclude tracts with low coverage in the dataset: only those present in at least 50% of the participants of each phenotype (CN, lvPPA, PCA, bvAD, and CBS) were included in further analysis. This conservative approach was intended both to reduce the likelihood of testing false-positive connections between brain areas and of conducting low-powered tests based on only a few participants.

As a further quality-control measure, we additionally restricted analysis to connections present in the HCP1065 diffusion template available on the DSI Studio website^3^, which was derived by averaging voxelwise ODFs for 1,065 young adult participants in the Human Connectome Project study (Yeh, 2022). We performed deterministic tractography on this group-average template using the same tracking parameters described above, then used the same composite atlas to create a 152 × 152 connection matrix. Zero-valued connections in this population-average connectivity matrix were excluded from further analysis.

These rules resulted in retention of 497 tracts for analysis, out of a possible 11,476 pairwise connections. Retained tracts were present in a mean of 80.5% (SD = 11.9) of participants’ connectomes. Cases with missing values for retained tracts were excluded on a tract-by-tract basis. Tract labels were defined in DSI Studio by performing tractography on the HCP1065 template between the corresponding GM endpoint labels, then using atlas-based tract recognition (Yeh, 2022) to derive the most probable label for each tract. Tract labels are reported for statistically significant findings in Supplementary Tables 1, 2. A minority of tracts detected in the current dataset were not present in atlas-based tracking. These connections may reflect sample-specific anatomical variations, or they may represent false positive connections, despite the quality control procedures described above.

Additionally, the DSI Studio generalized q-sampling imaging (GQI) pipeline from QSIPrep output voxelwise maps for diffusion microstructure metrics including fractional anisotropy (FA; based on a diffusion tensor fit); mean diffusivity (MD), which is typically hypothesized to increase when WM tracts degenerate, allowing less restricted diffusion; and isotropic water diffusion (i.e., a “free water” component), which may represent the presence of cerebrospinal fluid or edema in a voxel. We computed a global mean for each component within each participant’s WM by eroding the WM tissue mask by 2 voxels in all directions to reduce risk of partial volume effects from adjacent GM or CSF, then averaging each metric over all voxels in the resulting mask.

### Statistical methods

#### Missing or imputed data

MMSE scores were unavailable for 4 CN and 2 amnestic AD participants. For the 4 CN cases, MMSE scores were imputed as the mean of the remaining CN sample; and for the 2 amnestic AD cases, scores were imputed as the mean MMSE among all amnestic AD and non-amnestic AD patients. Additionally, disease duration was unavailable for one lvPPA patient (male, 67 years old) and was imputed as the mean disease duration among all other patients. To address potential bias introduced by these imputations, we omitted cases with imputed data from subset analyses reported below (*Subset analyses*, Supplementary Tables 5–7). To prevent extreme outlier values from influencing regression-based analyses, we truncated w-scores for both tractwise GFA and regional volume at 0.1 and 99.9% of their respective distributions.

#### Neuropsychological assessments

Cognitive and behavioral performance was assessed using available data from a number of standardized assessments (Table 1). For each assessment, we used linear regression models to assess phenotypic differences, covarying for age, sex, and education. Post-hoc tests were performed for tasks that exhibited a significant main effect of phenotype [ α =0.05, adjusted for false discovery rate (Benjamini and Hochberg, 1995)]; post-hoc Tukey’s tests were performed with a criterion of α =0.05.

**Table 1:** Neuropsychological performance by phenotype.

Phenotype	Animals	Boston naming test	Backward digit span	FAQ	Forward digit span	F words	JOLO	NPI-Q	Rey figure copy	Rey figure recall	PBAC total memory
Normal	45	21.0 [15.0–26.2] (28)	29.0 [27.0–30.0] (27)	6.0 [5.0–7.0] (19)	0.0 [0.0–0.0] (5)	7.0 [6.5–7.5] (19)	18.0 [15.0–19.5] (19)	6.0 [6.0–6.0] (12)	0.0 [0.0–0.0] (7)	36.0 [34.0–36.0] (15)	18.0 [15.0–22.8] (15)
aAD	41	8.0 [4.0–14.0] (37)	21.5 [14.0–26.0] (38)	3.0 [2.0–4.0] (34)	12.0 [7.0–20.0] (23)	6.0 [5.0–7.0] (33)	8.0 [4.0–13.5] (32)	5.0 [2.8–6.0] (24)	5.0 [2.0–6.0] (29)	29.0 [9.5–33.0] (19)	1.0 [0.0–6.0] (19)
lvPPA	32	10.0 [6.0–13.8] (30)	20.0 [11.0–26.5] (27)	3.0 [3.0–4.0] (25)	2.5 [0.0–6.2] (20)	4.0 [3.0–5.0] (25)	7.0 [4.0–10.0] (25)	5.5 [4.8–6.0] (20)	2.0 [0.0–4.0] (25)	32.0 [24.0–36.0] (15)	11.0 [7.2–19.0] (15)
PCA	17	8.0 [5.0–14.0] (17)	23.0 [16.0–25.0] (17)	3.0 [2.0–3.0] (14)	15.0 [7.0–22.0] (9)	5.0 [4.0–6.0] (15)	13.5 [10.2–15.2] (16)	0.0 [0.0–4.0] (15)	3.0 [2.0–5.0] (14)	7.5 [3.0–15.1] (10)	2.5 [2.0–4.0] (10)
bvAD	10	7.0 [6.0–8.0] (10)	17.0 [14.2–26.0] (10)	3.0 [3.0–4.0] (9)	22.5 [19.0–26.5] (4)	5.0 [4.0–5.0] (9)	7.0 [4.0–13.0] (9)	5.0 [4.5–6.0] (7)	8.0 [5.5–9.2] (8)	14.5 [9.8–21.5] (7)	4.5 [2.0–6.5] (7)
CBS	8	8.0 [6.5–9.0] (7)	16.0 [11.0–22.5] (7)	2.0 [2.0–2.5] (7)	13.5 [11.8–15.2] (2)	5.0 [4.5–5.0] (7)	4.0 [4.0–7.5] (7)	3.5 [3.0–5.5] (6)	4.0 [3.2–4.8] (6)	7.0 [3.8–10.2] (2)	7.8 [3.9–11.6] (2)
		**4.34e-12**	**1.15e-05**	**1.88e-10**	**1.18e-05**	**2.82e-07**	**1.44e-08**	**9.38e-05**	**1.70e-05**	**1.44e-07**	**5.39e-09**

Each cell reports the median, interquartile range, and number of observations. Boldface indicates statistically-significant *p*-values.

#### W-score transformations

GM volumes were pre-adjusted for associations with intracranial volume, age at MRI, and sex by conversion to w-scores (La Joie et al., 2013) based on regression models computed in CN participants. Similarly, mean GFA scores for WM tracts of interest were converted to w-scores, adjusting for age at MRI and sex effects. While w-scores were correlated with the untransformed GM volumes and tractwise GFA values, their interpretation differs: higher scores indicated greater volume or GFA than expected for a cognitively normal individual of the same age and sex, while lower scores indicated greater atrophy or WM degeneration than expected.

#### Tractwise contrasts

We aimed to identify both patterns of WM degeneration that characterized non-amnestic AD generally as well as those specific to lvPPA, PCA, CBS, and bvAD. We thus performed contrasted tractwise GFA values both by group (CN, amnestic AD, or non-amnestic AD) and by phenotype (CN, amnestic AD, lvPPA, PCA, CBS, or bvAD) using linear regression to perform mass univariate analyses of all 497 retained tracts. For each, we computed a model with w-scores for tractwise mean GFA as the dependent variable; group or phenotype as a fixed effect of interest; and MMSE score to adjust for within-group variation in disease progression. Post-hoc tests of group and phenotype were performed for tracts that exhibited a significant effect of either factor based on analysis of variance (ANOVA). Significance for ANOVAs was determined by a criterion of α =0.05, FDR-adjusted; post-hoc tests used α =0.05 after Tukey’s adjustment.

#### Associations between grey and white matter degeneration

To determine whether GM and WM degeneration were mutually associated, we first computed a mean WM w-score over all tracts projecting from a given region in each participant. This mean WM score served as the outcome variable in a linear mixed effects model with fixed effects of group, GM w-score, and the group × GM w-score interaction; and a second, parallel model with fixed effects of phenotype, GM w-score, and the phenotype × GM w-score interaction. Both models included a random intercept per participant to account for correlation due to within-subject repeated measures (i.e., multiple regions). Significance of model effects was assessed at α =0.05, uncorrected.

#### Global differences in WM microstructural metrics

GM atrophy could influence tractography results by making it more difficult to trace continuous diffusion pathways between brain areas. Additionally, in individuals with lower GM and WM volume, we reasoned that tractwise GFA could be underestimated due to partial-volume effects at the GM/WM border. To provide converging tests of group differences in WM degeneration, we thus used linear regression and analyses of variance to test for effects of group on mean FA, MD, and isotropic diffusion. Regression models included covariates of age, sex, and MMSE score; significance was assessed at α =0.05, FDR-corrected. Post-hoc Tukey’s tests were performed for metrics that indicated significant effects of group.

#### Topological features of brain networks using persistent homology

Topological analysis was performed on graphs defined both from untransformed tractwise GFA values and their corresponding w-scores. To simplify interpretation of tractwise connection strength, we performed a min/max normalization of WM w-scores, setting the global minimum value in the dataset equal to zero and the global maximum equal to one. Tracts with missing values were represented as zeros. Topological metrics included Betti-0 numbers, representing the number of components at a given threshold; Betti-1 numbers, indicating the number of cycles (i.e., loops) at that threshold; and the ratio of Betti-1 to Betti-0 (i.e., the ratio of cycles to components), where a large ratio indicates that a network is not maximally dense (Kartun-Giles and Bianconi, 2019), suggesting more efficient network architecture.

Next, graph filtration thresholds were computed using the sorted sequence of weights from the minimum spanning tree of each participant’s network (Lee et al., 2012) and 100 linearly spaced thresholds between the min and max weights across the participants. Betti-0 numbers were computed using the Dulmage-Mendelsohn decomposition (Dulmage and Mendelsohn, 1958; Pothen and Fan, 1990) of each sub-network. Betti-1 numbers were computed analytically using the Euler characteristic by considering only the algebraically independent loops (Lee et al., 2014) (i.e., Betti-1 = Betti-0 – # parcellations + # edges). The Betti curves are monotone over the graph filtrations, i.e., they are increasing for number of components and decreasing for number of loops.

The statistical significance of the difference between the mean Betti curves of groups was estimated using the Kolmogorov–Smirnov (KS) distance between them, which is the maximum distance between two Betti curves (Chung et al., 2019). *P*-values were computed similarly to the KS test (Kolmogorov, 1933; Smirnov, 1948) by mapping the Betti curves as walks on a Cartesian grid and enumerating every possible walk on the grids combinatorially (Chung et al., 2017). The size of the Cartesian grid is (q + 1) x (q + 1), where q is the maximum number of connected components (i.e., the number of nodes) for Betti-0 and the maximum number of cycles (estimated from Euler’s formula is proportional to the number of edges) for Betti-1.

We selected the max distance filtration threshold between the CN and non-amnestic AD groups and calculated the Betti numbers of the corresponding sub-network for all participants. Additionally, we investigated the clinical relevance of Betti-0, Betti-1, and the Betti-1/Betti-0 ratio through multiple regression models with MMSE score and disease duration as outcome variables. Predictors included each Betti metric plus age at MRI and sex as covariates. Significance was assessed at α =0.05, uncorrected. Supplementary Table 3 reports simple Pearson’s correlations of Betti numbers with WM microstructure metrics (FA, MD, and isotropic diffusion).

### Statistical software

**Figure 2:**
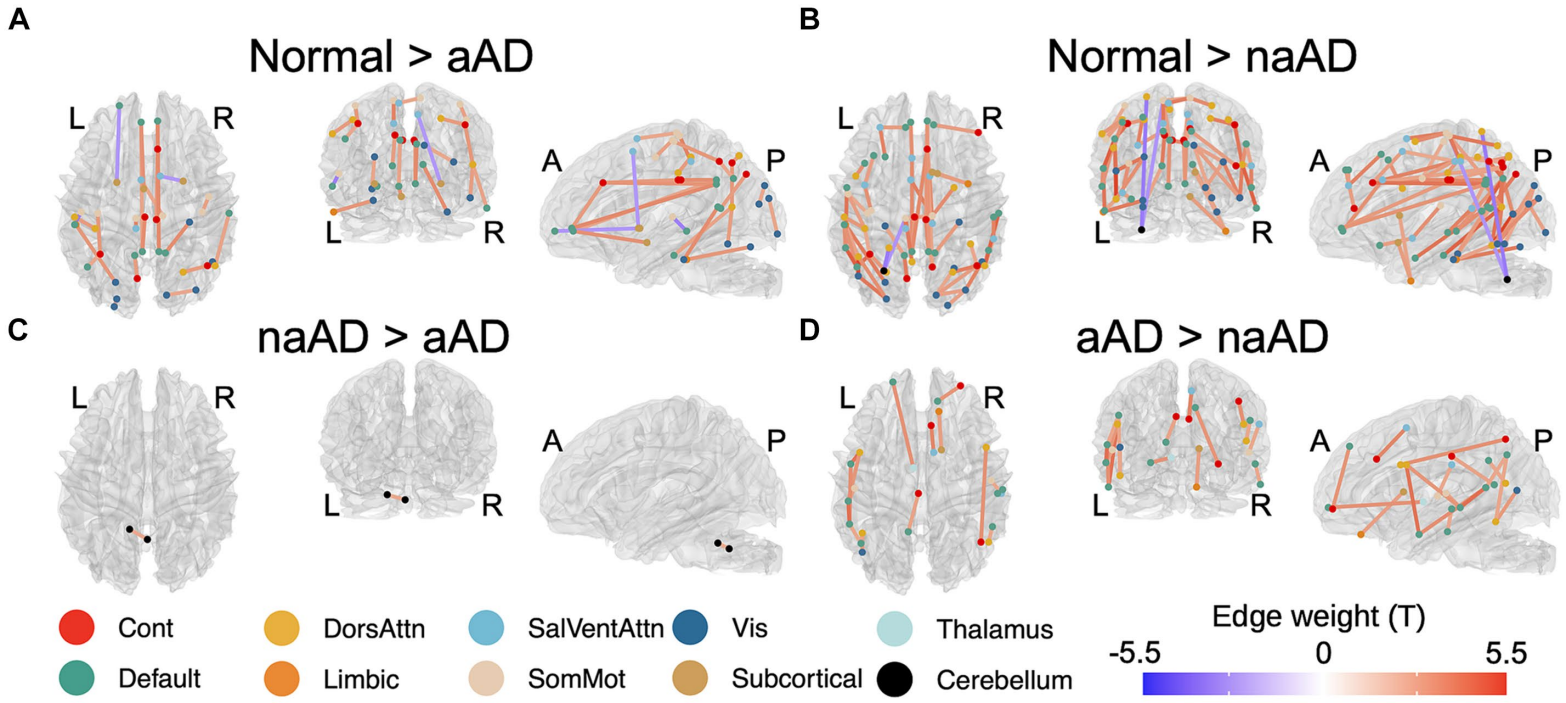
Significant between-group differences in tractwise w-scores (calculated as mean generalized fractional anisotropy, adjusting for age at MRI and sex). **(A)** Cognitively-normal - amnestic AD; **(B)** Cognitively normal - non-amnestic AD; **(C)** Amnestic AD > non-amnestic AD; **(D)** Non-amnestic AD > amnestic AD. Edge color represents t-statistics from post-hoc Tukey’s test for edges that exhibited a significant main effect of group in analyses of variance. Warm colors indicate higher w-scores for the first group than the second in each plot; cool colors indicate lower w-scores for the first group than the second.

**Figure 3:**
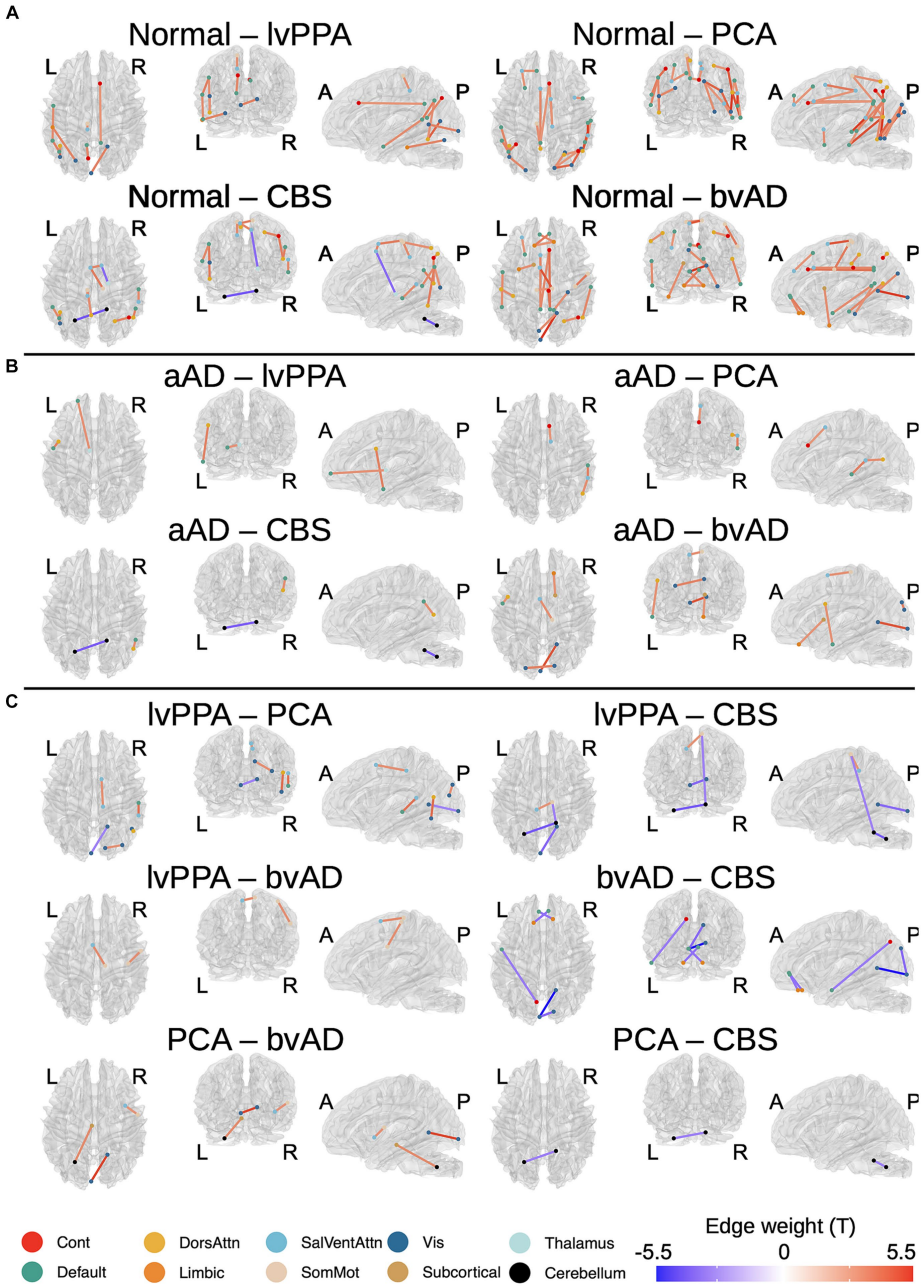
Differences in tractwise w-scores (calculated as mean generalized fractional anisotropy, adjusting for age at MRI and sex) between phenotypes: cognitively normal, amnestic AD (aAD), logopenic-variant primary progressive aphasia (lvPPA), posterior cortical atrophy (PCA), behavioral-variant AD (bvAD), and corticobasal syndrome (CBS). **(A)** Cognitively normal vs. each non-amnestic AD phenotype; **(B)** Amnestic AD vs. each non-amnestic AD phenotype; **(C)** contrasts between non-amnestic AD phenotypes. Edge color represents t-statistics from post-hoc Tukey’s tests for edges that exhibited a significant main effect of group or phenotype in analyses of variance. Warm colors indicate higher w-scores for the first group than the second in each plot; cool colors indicate lower w-scores for the first group than the second.

R version 4.3.1 and the R packages lmerTest (Kuznetsova et al., 2017), emmeans (Lenth, 2024), and stats (Wilkinson and Rogers, 1973; Chambers and Hastie, 1992) were used to perform tractwise contrasts, test associations between GM and WM degeneration, and correlate Betti numbers with clinical and microstructural variables. MATLAB was used to contrast the Kolmogorov–Smirnov distance between Betti profiles for different groups. Tractwise contrast results (Figures 2, 3) were created using the brainconn2 R package (Orchard et al., 2020; Mahzarnia et al., 2022). Source code used to generate figures and statistical calculations will be released on JSP’s GitHub.^4^

## Results

### Participant characteristics

An ANOVA indicated that age at MRI varied significantly by group [*F*(2,150) = 4.69, *p* = 0.01]: consistent with prior reports, non-amnestic AD cases were significantly younger at MRI than amnestic AD cases (*t* = −2.93, *p* = 0.004) and marginally younger than controls (*t* = −1.96, *p* = 0.05). The amnestic AD and control groups did not differ in age at MRI (*t* = −0.94, *p* = 0.35). MMSE score also differed by group [*F*(2,150) = 33.84, *p* = 7.42e-13]: both amnestic (*t* = 6.83, *p* = 2.03e-10) and non-amnestic patients (*t* = −7.53, *p* = 4.44e-12) had lower scores than controls but did not differ from each other (*t* = 0.11, *p* = 0.91). The amnestic and non-amnestic groups also did not differ in disease duration [*F*(1,106) = 0.01, *p* = 0.92]. We observed no group difference in sex distribution [ $\chi^{2}$ =0.72, *p* = 0.72]; however, the number of *APOE* ϵ4 alleles varied significantly [ $\chi^{2}$ =15.49, *p* = 0.004], reflecting group differences in the prevalence of risk allele carriers: 33.3% of controls and 41.9% of non-amnestic AD patients with available genotypes carried the ϵ4 allele, compared to 80.0% of amnestic AD cases.

Age also differed by phenotype (Table 2). Post-hoc Tukey’s tests showed that the CN group had a higher mean age than PCA (*t* = 2.16, *p* = 0.03). Additionally, the amnestic AD group were older than the CBS (*t* = 2.32, *p* = 0.02) and PCA (*t* = 2.84, *p* = 0.005) groups. The majority of amnestic AD participants (61.0%) had an estimated disease onset at age 65 or earlier. An additional 14.6% had an onset between 65 and 70 years of age, and the remaining amnestic AD patients had onset after age 70. In comparison, 82.1% of non-amnestic AD participants had an estimated onset of age 65 or earlier; 7.5% between 65 and 70; and 9.0% after age 70. In tests of global cognition, a highly significant group effect (Table 2) reflected significantly better cognition for the CN group than all patients (all *T* > 4.1, *p* < 0.0001); no significant differences were found between phenotypes (all *T* < 1.72, *p* > 0.08). The frequency of the *APOE* ∈4 allele differed by phenotype [ $\chi^{2}$ =42.7, *p* = 0.0005]: the ∈4 allele was more common in amnestic AD and bvAD, and less common in lvPPA and PCA (Table 2). No phenotypic differences in sex ratio were found, and patient groups did not significantly differ in disease duration.

**Table 2:** Participant characteristics.

		Normal	aAD	lvPPA	PCA	bvAD	CBS	*p*
N		45	41	32	17	10	8	
Age		64.0 [59.0–70.0]	67.0 [59.0–75.0]	63.5 [57.0–68.2]	57.0 [55.0–62.0]	60.5 [54.5–68.5]	57.5 [54.0–60.5]	**0.03**
MMSE Total		29.2 [29.0–30.0]	22.9 [20.0–25.0]	24.0 [19.0–27.2]	25.0 [19.0–26.0]	19.0 [16.0–23.0]	19.0 [15.0–24.0]	**1.37e-11**
Duration		0.0 [0.0–0.0]	4.0 [2.0–5.0]	3.0 [2.0–4.1]	3.0 [1.0–5.0]	3.5 [1.2–6.0]	4.0 [2.8–4.8]	**6.68e-17**
Sex	Female	26 (57.8)	20 (48.8)	16 (50.0)	12 (70.6)	2 (20.0)	5 (62.5)	0.18
	Male	19 (42.2)	21 (51.2)	16 (50.0)	5 (29.4)	8 (80.0)	3 (37.5)	
APOE ε4 allele count	0	8 (17.8)	6 (14.6)	20 (62.5)	10 (58.8)	5 (50.0)	1 (12.5)	**1.00e-04**
	1	2 (4.4)	18 (43.9)	11 (34.4)	4 (23.5)	5 (50.0)	2 (25.0)	
	2	2 (4.4)	6 (14.6)	0 (0.0)	3 (17.6)	0 (0.0)	1 (12.5)	
	N/A	33 (73.3)	11 (26.8)	1 (3.1)	0 (0.0)	0 (0.0)	4 (50.0)	

For age, MMSE, and disease duration, each cell reports the median and interquartile range. For sex and APOE ε4 allele count, each cell reports the number (percentage) of individuals. Boldface indicates statistically-significant *p*-values.

### Neuropsychological performance

Cognitive and behavioral features for each phenotype are detailed in Table 1. For all assessments, the main effect of phenotype was significant (all *F* > 4.0, *p* < 0.0025). Post-hoc tests (Supplementary Table 1) indicated that patients were impaired on most tasks relative to the CN group, with exceptions. The amnestic AD group did not differ from the CN group in forward digit span. The lvPPA group did not differ from controls in copying and delayed recall of the Rey complex figure or on the Functional Activities Questionnaire (FAQ). The CBS group also did not differ from controls in delayed recall of the Rey figure or on the FAQ; however, these results cannot be reliably interpreted due to the low number of observations available in the CBS group. Notably, the PCA group was the only patient group to exhibit impairment on the Judgment of Line Orientation (JOLO) task; and the amnestic AD and bvAD groups were the only groups with significantly higher symptom counts than controls on the brief form of the Neuropsychiatric Inventory (NPI-Q) (Kaufer et al., 2000).

In contrasts between patient groups, the lvPPA group had significantly higher performance than PCA patients on the JOLO and Rey figure copy (Supplementary Table 1). LvPPA patients also had a higher total memory score on the Philadelphia Brief Assessment of Cognition (PBAC) than the amnestic AD and PCA groups; and superior recall of the Rey figure than the PCA, amnestic AD, and bvAD groups. In contrast, lvPPA patients had lower forward digit spans than the amnestic AD group; and lower scores on the F-words tasks than the PCA group. LvPPA patients additionally exhibited less impairment in functional activities than the amnestic AD, PCA, and bvAD groups. Finally, the bvAD group had a greater frequency of neuropsychiatric symptoms than the amnestic AD, lvPPA, and PCA groups.

### Tract-wise differences by group and phenotype

A total of 164 tracts out of 497 tested exhibited significant differences between the 3 groups (CN, amnestic AD, and non-amnestic AD). Post-hoc Tukey’s tests indicated that both the amnestic and non-amnestic AD groups had widespread WM degeneration relative to the CN group (Figures 2A,B). Affected tracts in both groups included bilateral fronto-parietal and parahippocampal-parietal segments of the cingulum; the superior longitudinal fasciculi bilaterally; the right parietal aslant tract; and the body of the corpus callosum. Overall, amnestic AD patients exhibited lower WM w-scores than controls in 22 tracts, while non-amnestic AD patients had lower scores than controls in 61 tracts. In direct comparisons between the amnestic and non-amnestic AD groups, amnestic AD patients had lower WM scores than non-amnestic AD patients only in one intracerebellar tract (Figure 2C). In the left hemisphere, non-amnestic AD patients exhibited lower WM scores than amnestic AD patients (Figure 2D) in the arcuate and superior and inferior longitudinal fasciculi; frontal-parahippocampal segment of the cingulum; and the parietal aslant tract. In the right hemisphere, non-amnestic AD patients differed from amnestic AD in the inferior fronto-occipital fasciculus and superior longitudinal fasciculus I and II. Finally, non-amnestic AD patients had specific WM degeneration in the forceps minor.

We observed a significant effect of phenotype (CN, amnestic AD, lvPPA, PCA, CBS, or bvAD) in 113 of the tracts that exhibited group differences, plus an additional 21 connections. A complete list of significant post-hoc findings is included in Supplementary Table 2. We note that contrasts involving bvAD and CBS participants should be interpreted with caution due to the lower sample sizes in these groups; we report them in Figure 3 for the sake of transparency. Consistent with the left posterior temporal epicenter of disease in lvPPA (Phillips et al., 2018a), patients exhibited WM degeneration relative to controls in several tracts connecting left temporal nodes of the default-mode network, including left arcuate and inferior longitudinal fasciculi; left superior longitudinal fasciculus I; and the left parietal aslant tract. Additional findings involved bilateral cingulum and the forceps major. PCA participants exhibited a bilateral, mostly posterior pattern of WM atrophy that principally involved tracts in the dorsal attentional, salience, and visual networks, in accordance with the visuospatial, constructional, and executive function deficits observed in PCA. Affected tracts included bilateral parietal aslant tracts; vertical occipital, inferior longitudinal, and superior longitudinal fasciculi; and the frontal–parietal segments of the cingulum. Left-lateralized degeneration was observed in the frontal aslant tract. While CBS exhibited an overlapping pattern of WM degeneration with PCA (including the parietal aslant tracts and right superior longitudinal fasciculus), there were fewer differences from the CN group overall and less involvement of visual network connections. However, CBS also exhibited lower WM scores than controls in left cerebellum, left cingulum, and the body of the corpus callosum. The bvAD group exhibited degeneration of bilateral midline and interhemispheric WM tracts including the callosal body, forceps major, and forceps minor; bilateral cingulum and superior longitudinal fasciculi; left arcuate fasciculus; and right inferior longitudinal fasciculus.

Pairwise phenotypic contrasts further highlighted differences between amnestic and non-amnestic AD (Figure 3B). Amnestic AD had higher WM scores than lvPPA in fibers connecting left anterior temporal cortex with the left frontal eye fields, as well as in fibers connecting left prefrontal cortex with the anterior thalamus. Amnestic AD patients also had higher WM scores than PCA patients in the right superior longitudinal fasciculus I; and higher scores than both PCA and CBS patients in the right parietal aslant tract. Conversely, CBS patients had less WM degeneration than aAD patients in left cerebellar WM. BvAD patients showed reduced WM integrity vs. amnestic AD in left-hemisphere tracts connecting temporal cortex with the frontal eye fields; right inferior fronto-occipital fasciculus; the forceps major, and the body of the corpus callosum.

A comparison of the two largest non-amnestic AD groups (Figure 3C), lvPPA and PCA, indicated that lvPPA patients had higher WM scores in right cingulum, parietal aslant tract, and inferior longitudinal fasciculus, consistent with differential disease lateralization in the two syndromes; however, PCA patients did not have correspondingly higher WM scores than the lvPPA group in the left hemisphere. LvPPA patients also had lower WM integrity than the PCA group in a portion of the forceps major connecting left posterior occipital cortex with right anterior occipito-temporal cortex. LvPPA patients had less WM degeneration than bvAD patients in callosal fibers connecting left medial frontal and right somatomotor cortex, as well as in a portion of right superior longitudinal fasciculus II connecting portions of somatomotor cortex. The lvPPA group had less degeneration than CBS patients in projections from right somatomotor cortex to right cerebellum and to left medial prefrontal cortex; as well as in tracts connecting the left and right nodes of the cerebellum. PCA patients exhibited stronger WM integrity than the bvAD group in the right corticostriatal tract and in fibers connecting the left cerebellum and red nucleus. Contrary to expectations, the bvAD group had lower WM integrity than the PCA and CBS groups in a segment of the forceps major connecting left and right anterior visual regions; and in the forceps minor and left precuneus-temporal projections relative to the CBS group.

### Differences in global white matter microstructure

**Figure 4:**
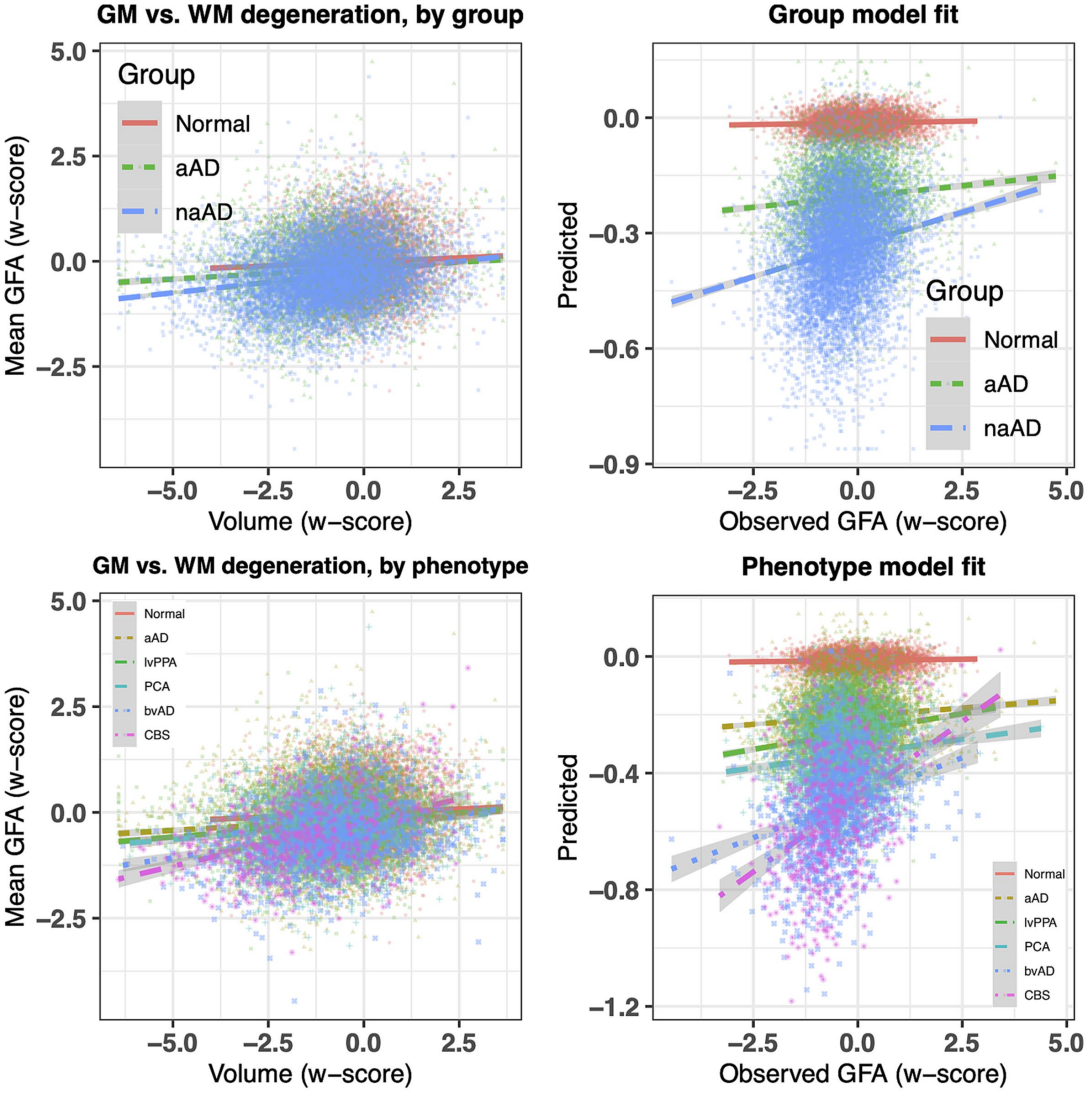
Between-group contrasts of global fractional anisotropy, mean diffusivity, and isotropic diffusion, averaged for each individual over voxels in an eroded white matter mask. The non-amnestic AD group had lower global FA (*p* = 0.0017), higher mean diffusivity (*p* = 0.030), and marginally higher isotropic diffusion (*p* = 0.051) than the amnestic AD group, covarying for age, sex, and MMSE score.

To provide converging evidence for syndromic differences in WM degeneration, we next tested whether mean values of FA, MD, and isotropic diffusion (averaged across voxels in an eroded WM mask for each participant) differed by group (Figure 4). All 3 metrics showed a significant effect of group: FA, *F*(2,146) = 7.24, *p* = 0.001; MD, *F*(2,146) = 3.44, *p* = 0.03; and isotropic diffusion, *F*(2,146) = 3.51, *p* = 0.03. In post-hoc tests, amnestic AD patients had higher FA than non-amnestic AD patients [*t*(146) = 3.51, *p* = 0.002] as well as lower MD [*t*(146) = −2.57, *p* = 0.03]. Isotropic diffusion was also marginally lower in amnestic than non-amnestic AD [*t*(146) = −2.36, *p* = 0.05].

### Associations between grey and white matter degeneration

**Figure 5:**
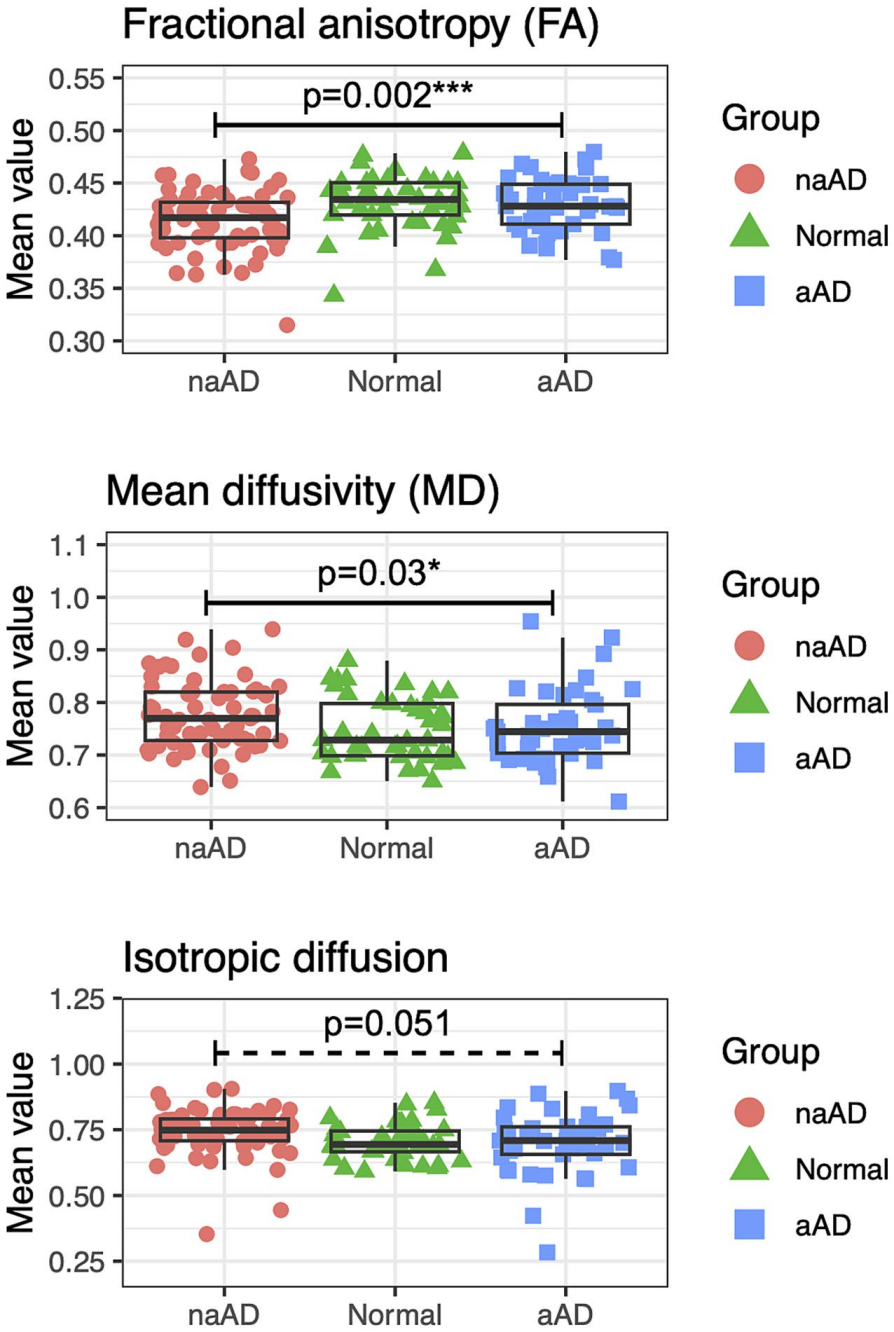
Top left: association of regional atrophy with mean WM w-scores for tractwise generalized fractional anisotropy (GFA), stratified by group (normal, amnestic, and non-amnestic). Top right: observed mean GFA w-scores vs. fixed-effects model fits. Bottom left: regional atrophy vs. mean GFA w-scores, stratified by phenotype (normal, amnestic MCI/AD, lvPPA, PCA, bvAD, and CBS). Bottom right: observed observed mean GFA values for each region vs. fixed-effects fits (bottom).

We next investigated whether associations between WM and GM degeneration varied by group and phenotype. Table 3 reports fixed effects for the group model. An ANOVA on this model indicated a main effect of GM volume [*F*(1,20,442) = 326.02, *p* = 2.58e-72] and an interaction of group × volume [*F*(2,20,443) = 27.60, *p* = 1.07e-12]. This interaction reflected a difference in the slope of association between GM and WM w-scores for the three groups (Figure 5, top left), with significantly more positive slopes for the amnestic and non-amnestic AD groups relative to the CN group (Table 3). Fixed effects fits explained 4.63% of the variance in mean WM w-scores. The phenotype model (Table 4) similarly indicated a main effect of GM w-score [*F*(1,20,436) = 470.40, *p* = 3.81e-103] and a significant phenotype × GM w-score interaction [*F*(5,20,437) = 14.27, *p* = 5.64e-14], with greater positive slopes for the 5 patient groups relative to controls (Table 3; Figure 5, bottom left). This model accounted for 5.36 percent of the variance in mean WM scores.

**Table 3:** Associations of mean white matter w-scores with group, regional GM volume w-scores, and group x volume interactions.

Term	Coefficient	Std. error	Statistic	DF	Value of *p*
(Intercept)	−0.014	0.080	−0.170	146	0.8650
GroupaAD	−0.121	0.116	−1.044	147	0.2981
GroupnaAD	−0.242	0.105	−2.302	146	**0.0228**
WVol	0.026	0.008	3.340	20,437	**0.0008**
GroupaAD:WVol	0.051	0.010	5.106	20,439	**0.0000**
GroupnaAD:WVol	0.068	0.009	7.430	20,442	**0.0000**

Boldface indicates statistically-significant *p*-values.

**Table 4:** Associations of mean white matter w-scores with phenotype, regional volume, and group x phenotype interactions.

Term	Coefficient	Std. error	Statistic	DF	Value of *p*
(Intercept)	−0.014	0.080	−0.170	143	0.8654
PhenotypeaAD	−0.121	0.116	−1.042	144	0.2994
PhenotypelvPPA	−0.184	0.125	−1.474	144	0.1427
PhenotypePCA	−0.239	0.154	−1.556	144	0.1218
PhenotypebvAD	−0.398	0.189	−2.111	144	**0.0365**
PhenotypeCBS	−0.323	0.282	−1.144	145	0.2544
WVol	0.026	0.008	3.341	20,433	**0.0008**
PhenotypeaAD:WVol	0.051	0.010	5.108	20,435	**0.0000**
PhenotypelvPPA:WVol	0.052	0.010	5.050	20,452	**0.0000**
PhenotypePCA:WVol	0.068	0.012	5.583	20,424	**0.0000**
PhenotypebvAD:WVol	0.092	0.013	6.845	20,422	**0.0000**
PhenotypeCBS:WVol	0.105	0.017	6.024	20,442	**0.0000**

Boldface indicates statistically-significant *p*-values.

In the subset of 104 participants with known *APOE* genotype, we calculated a similar model with fixed effects of *APOE* ∈4 copy number (0, 1, or 2), GM w-score, and their interaction; as well as a random intercept per participant. This model yielded a null effect of *APOE* genotype [*F*(2,102) = 0.43, *p* = 0.65] but a significant main effect of GM volume [*F*(1,14,291) = 318.02, *p* = 2.26e-70] and a significant interaction of *APOE* ∈4 with GM volume [*F*(2,14,293) = 3.86, *p* = 0.02]. This interaction reflected the fact that people with zero copies of the ∈4 allele had a steeper slope of association between WM and GM w-scores than individuals with one [ β = − 0.020, *t* = −2.48, *p* = 0.01] or two [ β = − 0.024, *t* = −1.93, *p* = 0.05] copies. Because *APOE* genotype was partially confounded with diagnostic group, we calculated two additional models: one with additive effects of group (CN, amnestic AD, or non-amnestic AD), *APOE* ∈4 copy number, and GM w-scores; and a second testing main effects and all two- and three-way interactions of these factors. When the factor of group was included, neither the main effect of *APOE* ∈4 nor any of its interactions with other factors were significant (all *F* < 1.01, *p* > 0.36).

### Global differences in network topology

**Figure 6:**
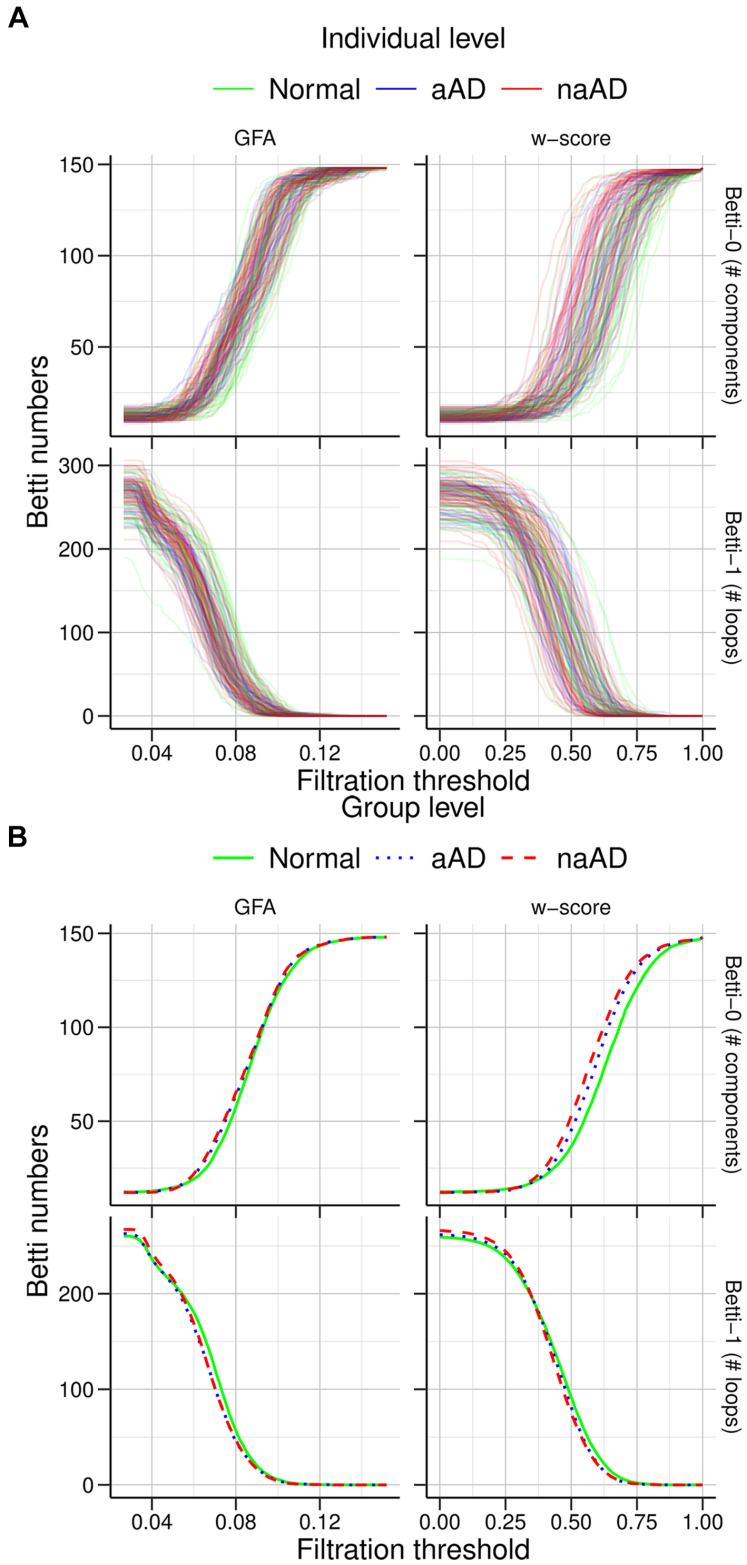
Graph filtration results for cognitively normal, amnestic AD, and non-amnestic AD groups. **(A)** Individual filtration curves for graphs based on generalized fractional anisotropy (GFA) values (left) and w-score-transformed GFA values (right). The top row shows increasing Betti-0 numbers with stricter thresholds, while the bottom row shows fewer cycles/loops as the threshold increases. **(B)** Mean curves for each group. The non-amnestic AD group exhibited significantly higher Betti-0 numbers than the cognitively normal group, indicating more graph components; no other group differences were observed.

The individual and mean Betti curves are shown in Figure 6. For all participants, the number of components (Betti-0) necessarily increased at higher filtration thresholds, indicating more disconnected sub-networks in the brain as fewer edges survived the threshold. Over these different filtration thresholds, the number of components in the brain was consistently higher in non-amnestic AD than in the CN group (*p* < 0.05), though it did not differ between amnestic AD and CN or between amnestic and non-amnestic AD (both *p* > 0.05). Conversely, at higher thresholds the number of cycles (Betti-1) was reduced, as the number of traversable edges fell. While Betti-1 curves appeared to decline more steeply for the amnestic and non-amnestic AD groups than for CN participants, no significant group differences were observed (all *p* > 0.05).

### Topological metric associations with global cognition and disease duration

**Figure 7:**
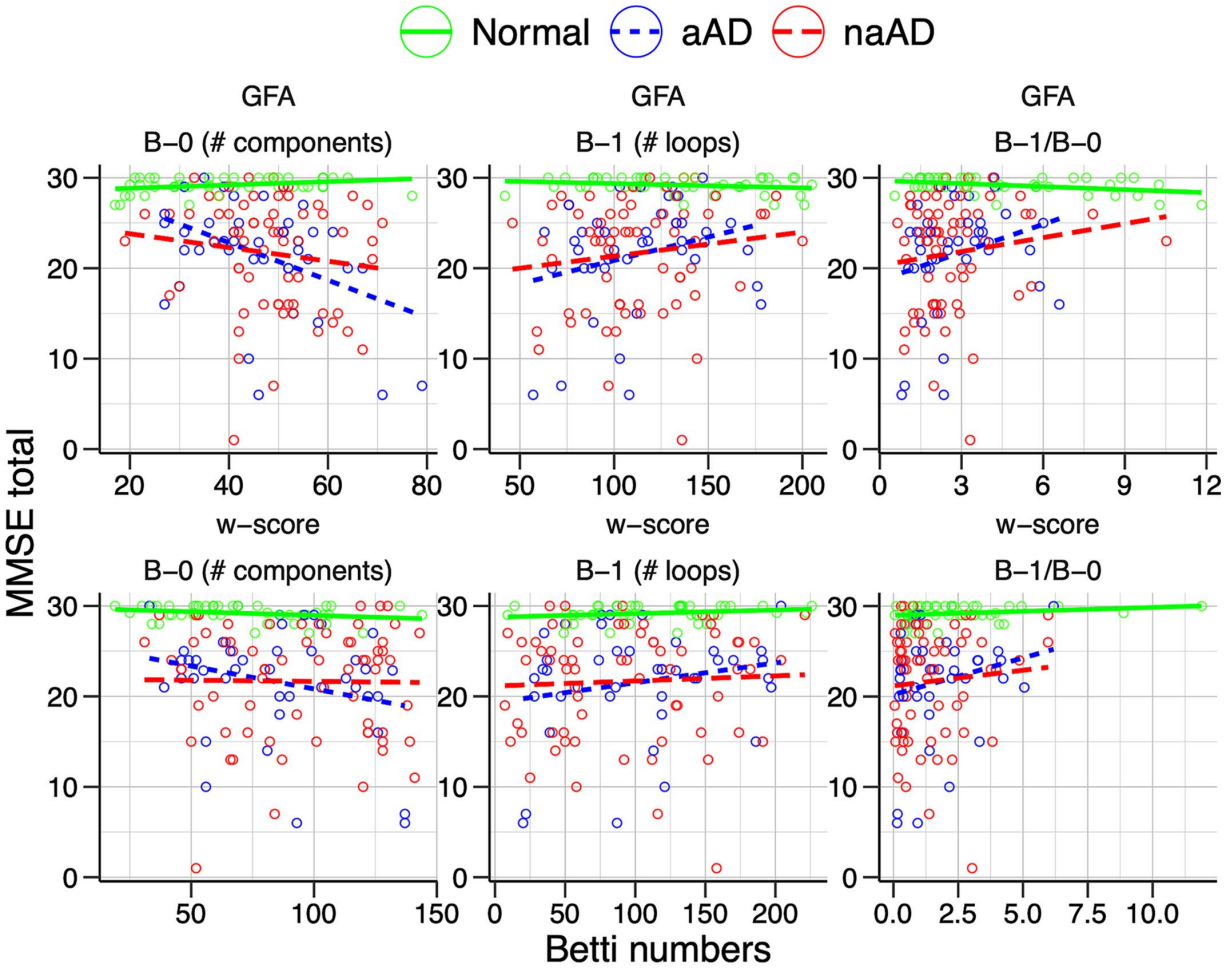
Associations of individuals’ Betti-0 (B-0) and Betti-1 (B-1) numbers as well as the Betti-1/Betti-0 ratio with total MMSE score. Top: Betti numbers were calculated from graphs using untransformed generalized fractional anisotropy (GFA) values. Bottom: Betti numbers based on graphs of w-score-transformed GFA values.

We additionally assessed associations of Betti numbers with MMSE and disease duration to gauge their sensitivity to disease severity. For graphs based on untransformed GFA values, Betti-0 was negatively associated with MMSE [ β = − 0.136, *t*(149) = −3.72, *p* = 0.0003], indicating global cognitive impairment was associated with greater disconnection of brain areas. Figure 7 (top left) illustrates this relationship with separate trend lines per group, suggesting it was driven by the amnestic and non-amnestic AD participants. Conversely, the Betti-1 model for GFA-based graphs (Figure 7, top middle) exhibited a positive association between Betti-1 and MMSE [ β =0.043, *t*(149) = 3.20, *p* = 0.002], suggesting that better global cognition was associated with greater frequency of loops in patients’ brain graphs. The Betti-1/Betti-0 ratio score (Figure 7, top right) had a similar positive association [ β =0.718, *t*(149) = 3.30, *p* = 0.001]. Betti numbers calculated from w-score-based graphs (Figure 7, bottom row) had similar, statistically significant associations with MMSE as numbers based on untransformed GFA values (Table 5).

**Table 5:** Results of linear regressions of MMSE score on Betti metrics.

Graph type	Betti metric	Term	Coefficient	Std. error	T-statistic	Value of *p*
GFA	B-0 (# components)	(Intercept)	−0.136	0.037	−3.717	**0.0003**
		value	0.037	0.059	0.625	0.5327
		Age	−0.961	0.974	−0.987	0.3254
		SexMale	15.544	4.265	3.645	**0.0004**
GFA	B-1 (# loops)	(Intercept)	0.043	0.014	3.195	**0.0017**
		value	0.056	0.060	0.934	0.3516
		Age	−0.907	0.985	−0.921	0.3587
		Sex Male	26.720	4.175	6.400	**0.0000**
W-score	B-0 (# components)	(Intercept)	−0.049	0.015	−3.171	**0.0018**
		Value	0.030	0.060	0.507	0.6129
		Age	−1.396	0.998	−1.398	0.1641
		SexMale	19.952	3.990	5.000	**0.0000**
W-score	B-1 (# loops)	(Intercept)	0.018	0.009	1.986	**0.0489**
		Value	0.041	0.061	0.667	0.5056
		Age	−1.165	1.014	−1.148	0.2526
		SexMale	19.011	3.900	4.874	**0.0000**
GFA	B-1/B-0	(Intercept)	0.718	0.217	3.304	**0.0012**
		Value	0.046	0.060	0.774	0.4402
		Age	−0.918	0.983	−0.934	0.3519
		SexMale	20.940	3.878	5.399	**0.0000**
W-score	B-1/B-0	(Intercept)	0.760	0.284	2.672	**0.0084**
		Value	0.034	0.061	0.568	0.5708
		Age	−1.320	1.008	−1.310	0.1921
		Sex Male	−0.136	0.037	−3.717	**0.0003**

Boldface indicates statistically-significant *p*-values.

**Figure 8:**
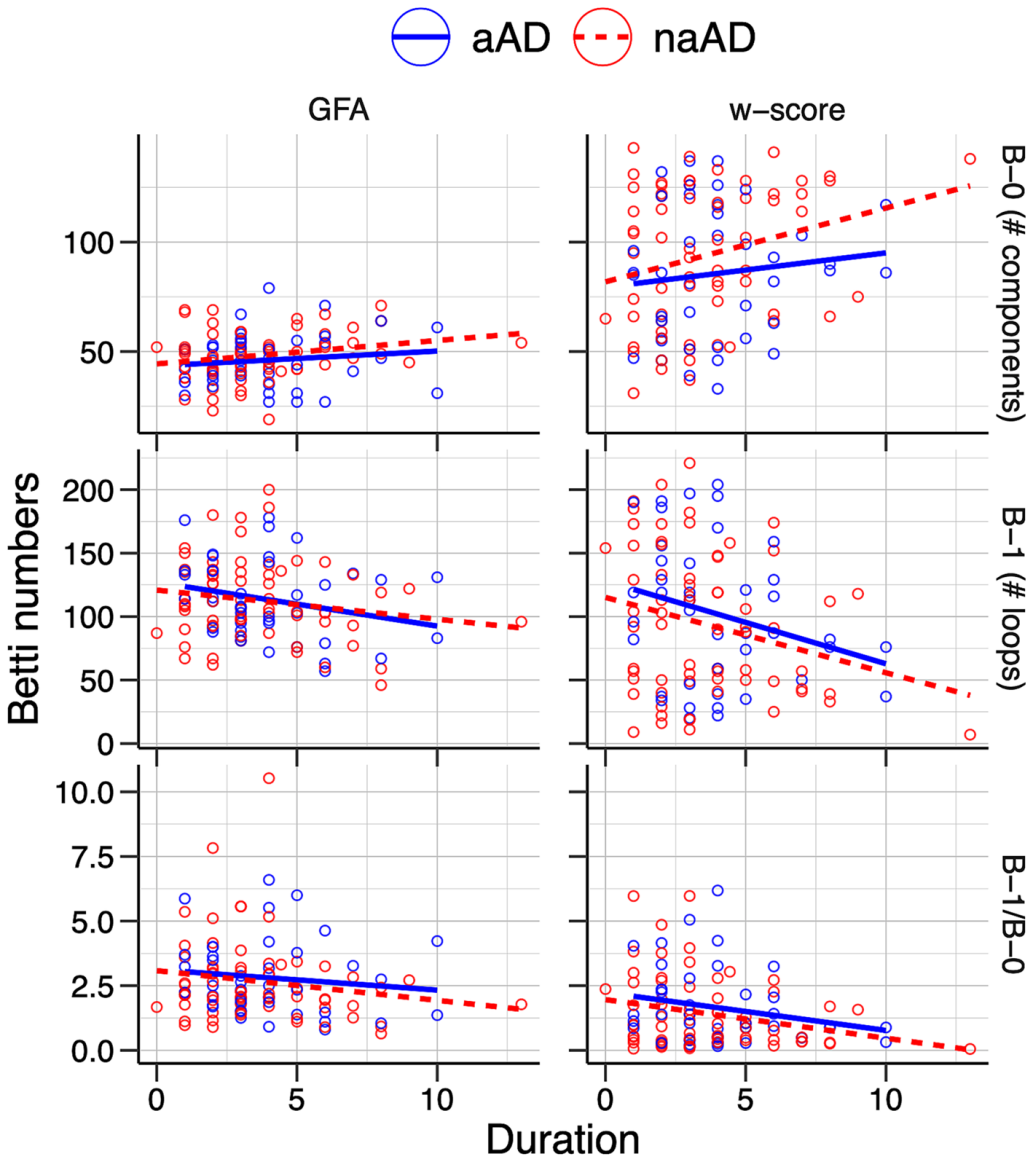
Associations of individuals’ Betti-0 (B-0) and Betti-1 (B-1) numbers as well as the Betti-1/Betti-0 ratio with estimated disease duration in the amnestic and non-amnestic AD groups. Left: Betti numbers were calculated from graphs using untransformed generalized fractional anisotropy (GFA) values. Right: Betti numbers based on graphs of w-score-transformed GFA values.

Relationships between Betti numbers and disease duration are illustrated in Figure 8; model results are given in Table 6. These models included only amnestic and non-amnestic AD participants, since disease duration was unknown for CN participants. As with MMSE, statistical models tested for a main effect of each Betti metric across groups; plots show separate trend lines per group to provide complementary information. Disease duration was positively associated with Betti-0 numbers for GFA-based graphs [ β =0.045, *t*(104) = 2.35, *p* = 0.02] and negatively associated with Betti-1 [ β = − 0.015, *t*(104) = −2.09, *p* = 0.04], though only marginally associated with the Betti-1/Betti-0 ratio [ β = − 0.243, *t*(104) = −1.73, *p* = 0.09]. For w-score-based graphs, the association with Betti-0 was non-significant [ β =0.016, *t*(104) = 2.29, *p* = 0.02], but Betti-1 [ β = − 0.011, *t*(104) = −2.77, *p* = 0.007] and the Betti ratio score [ β = − 0.405, *t*(104) = −2.61, *p* = 0.01] were both negatively associated with disease duration, indicating that disease progression was associated with a drop in the number of intact circuits between brain areas.

**Table 6:** Results of linear regressions of disease duration on Betti metrics.

Graph type	Betti metric	Term	Coefficient	Std. error	T-statistic	Value of *p*
GFA	B-0 (# components)	(Intercept)	−3.328	1.966	−1.693	0.0934
		Value	0.045	0.019	2.345	**0.0209**
		Age	0.077	0.026	3.001	**0.0034**
		Sex Male	0.049	0.441	0.110	0.9123
GFA	B-1 (# loops)	(Intercept)	0.988	1.814	0.545	0.5872
		Value	−0.015	0.007	−2.090	**0.0391**
		Age	0.069	0.026	2.687	**0.0084**
		Sex Male	−0.017	0.443	−0.038	0.9698
W-score	B-0 (# components)	(Intercept)	−2.403	1.780	−1.350	0.1799
		Value	0.016	0.007	2.290	**0.0241**
		Age	0.071	0.026	2.776	**0.0065**
		Sex Male	0.239	0.453	0.528	0.5984
W-score	B-1 (# loops)	(Intercept)	0.315	1.641	0.192	0.8480
		Value	−0.011	0.004	−2.773	**0.0066**
		Age	0.068	0.025	2.705	**0.0080**
		Sex Male	0.222	0.443	0.500	0.6179
GFA	B-1/B-0	(Intercept)	−0.199	1.660	−0.120	0.9049
		Value	−0.243	0.141	−1.731	0.0864
		Age	0.072	0.026	2.777	**0.0065**
		Sex Male	0.033	0.446	0.075	0.9406
W-score	B-1/B-0	(Intercept)	−0.090	1.623	−0.055	0.9559
		Value	−0.405	0.155	−2.614	0.0103
		Age	0.068	0.025	2.662	**0.0090**
		Sex Male	0.261	0.449	0.581	0.5626

Boldface indicates statistically-significant *p*-values.

### Subset analyses

We repeated tractwise contrasts and topological analyses omitting those patients without biomarker confirmation of AD pathologic change, as well as those with imputed MMSE scores and disease duration; these omissions left a subset of *n* = 139 participants. In tractwise contrasts, a total of 488 connections were included in both the full-dataset and the *n* = 139 subset analysis. Seven connections included in the full analysis did not satisfy our “50% rule” in the *n* = 139 subset; 10 connections not analyzed in the full analysis were included in the subset analysis. F-statistics for the main effect of group (amnestic AD, non-amnestic AD, or control) were highly correlated between the full dataset and subset analyses [*R*(486) = 0.972, *p* < 0.0001]. The main effect of phenotype (amnestic AD, lvPPA, PCA, bvAD, CBS, or control) was similarly highly consistent [*R*(486) = 0.978, *p* < 0.0001]. Of the 488 tracts in common between the full and subset analyses, significance of the group effect was concordant for 445 tracts (91.2%) at an FDR-adjusted alpha level of 0.05. Significance for the main effect of phenotype was concordant for 459/488 tracts (94.1%). Discordant results between the full and subset analyses are highlighted in Supplementary Table 5. In topological analyses, the statistical significance of results did not change for either the groupwise persistent homology comparisons (Supplementary Table 6) or the MMSE correlations (Supplementary Table 7). Associations between Betti metrics and disease duration also remained significant and highly consistent; the only exception was that the association between disease duration and Betti-0 number in the full analysis (*p* = 0.0241) became marginally significant in the subset analysis (*p* = 0.0665).

## Discussion

The present study asked whether individuals with non-amnestic AD syndromes exhibited similar or distinct patterns of WM degeneration as people diagnosed with amnestic MCI/AD. Furthermore, we tested whether observed variation in WM integrity, assessed by tractwise GFA, was related to patterns of GM degeneration in tract endpoints. We found that both amnestic and non-amnestic AD groups displayed lower GFA than CN participants in key fiber tracts including the cingulum, corpus callosum, superior longitudinal fasciculi, and others. Non-amnestic syndromes were characterized by more severe and widespread WM degeneration than in amnestic AD; and patients displayed phenotypic differences that corresponded with previously reported differences in GM degeneration (Ossenkoppele et al., 2015a; Phillips et al., 2018a). Differential breakdown of structural connectivity was corroborated by contrasts of global FA, MD, and isotropic diffusion; as well as by analysis of topological features, which indicated that non-amnestic AD patients had persistently greater fragmentation of brain graphs than CN participants. Topological metrics including Betti-0 (number of graph components), Betti-1 (number of loops/cycles), and the Betti-1/Betti-0 ratio were correlated both with clinical variables (MMSE and disease duration) and DWI microstructural metrics, supporting their utility as markers of cognitive and brain change. Finally, regional GM volumes had modest but highly significant associations with the mean GFA of fiber tracts projecting from each region, suggesting that WM degeneration in non-amnestic AD is at least partially related to GM disease progression, although the nature of this relationship remains uncertain.

Our findings corroborate and add to prior white matter imaging studies in early-onset amnestic and non-amnestic AD. We replicate Sintini et al. (2018)‘s findings of FA reductions in the splenium, cingulum, and posterior thalamic radiation in atypical AD (lvPPA and PCA); however, the current study adds greater context through comparisons to CN and amnestic AD groups; and anatomical detail through tractwise analysis. Caso et al. (2015) reported results from 28 early-onset amnestic AD, 12 lvPPA, and 13 PCA cases; as in this study, they found overlapping degeneration between amnestic and non-amnestic AD in the corpus callosum, cingulum, and superior longitudinal fasciculus. However, Caso et al. did not detect WM tract degeneration specific to lvPPA or PCA. The current study, based on a larger and more phenotypically-varied sample, found that PCA patients had greater WM degeneration than the lvPPA group in right-hemisphere occipital, temporal, and parietal projections; and all non-amnestic AD phenotypes exhibited unique differences relative to amnestic AD and CN participants. Additionally, we found that non-amnestic AD patients had greater WM degeneration than a sample of primarily early-onset amnestic patients; given that WM burden is greater in early-onset than late-onset amnestic AD (Sirkis et al., 2022), WM changes are likely to be even greater between non-amnestic AD and late-onset amnestic patients.

Several aspects of the findings from tractwise contrasts are consistent with hypothesized WM-mediated spread of disease from established, syndrome-specific GM epicenters. In lvPPA, the majority of tracts exhibiting degeneration were underlying left temporal and inferior parietal cortex, the earliest site of GM degeneration (Phillips et al., 2018a) and a peak area of tau accumulation (Phillips et al., 2021) in logopenic patients. In the PCA group, differences from controls included projections from posterior parietal, posterior temporal, and occipital cortex. This distribution was bilateral but included larger effect sizes in the right then left hemisphere, echoing previously reported asymmetry of GM atrophy effects (Phillips et al., 2019; Groot et al., 2020) and indicating the presence of PCA cases with greater right than left hemisphere involvement in the current sample. The PCA group additionally showed significant degeneration in frontoparietal segments of the cingulum and in projections from nodes in the dorsal attentional network, consistent with the longitudinal spread of disease from posterior to prefrontal brain areas over time (Andrade et al., 2013; Phillips et al., 2019; Katsumi et al., 2022). BvAD patients had reduced GFA in projections from limbic areas including orbitofrontal cortex and the anterior temporal lobes, as well as prefrontal nodes of the default mode and control networks. These sites are close to our previously reported epicenters for bvAD in left lateral prefrontal, left insular, and right middle temporal cortex (Phillips et al., 2018a) and are consistent with the hallmark deficits in affective processing, social behavior, and executive function that characterize bvAD (Ossenkoppele et al., 2022). [Prior research has found heterogeneous distributions of disease, indexed by tau positron emission tomography, in bvAD (Singleton et al., 2020)]. Finally, while results in the CBS group were likely underpowered and should be interpreted with caution, we observed a distributed pattern of WM degeneration encompassing left cingulum, the body of the corpus callosum, bilateral parietal aslant tracts, and the right superior longitudinal fasciculus. These tracts are located adjacent to previously reported epicenters of GM disease observed in CBS, including the left angular and supramarginal gyri as well as bilateral superior parietal lobules (Phillips et al., 2018a; Saito et al., 2022). Notably, WM differences between CBS and controls involved bilateral nodes of the somatomotor network (Supplementary Table 2), presenting possible WM correlates of the sensorimotor deficits observed in CBS (Armstrong et al., 2013).

### Associations between grey and white matter degeneration

Per hypotheses, we observed associations in WM tract integrity and GM atrophy that supported a link between degeneration in both tissue types. Observed correlations between WM and GM degeneration were small (on the order of 5% of variance explained by fixed-effects model fits). Interpreting the magnitude of these effects is difficult, as relatively few studies report quantitative associations between tractwise WM measures and atrophy of the corresponding GM endpoints. (Qualitative comparisons of GM and WM degeneration or correlations of GM atrophy with voxelwise WM microstructure metrics are more common). However, one recent study based on connectomic imaging from 2,789 datasets (Schilling et al., 2023) reported correlations of similar magnitude between FA and cortical thickness in older adults, in line with the current findings. The relatively modest association between GM and WM degeneration may indicate that both GM atrophy and WM degeneration are driven not only by some common processes but also tissue-specific factors.

One potential concern with tract-based analyses is that degeneration might reduce the ability of fiber-tracking algorithms to detect real WM tracts, as partial-volume effects from adjacent GM or cerebrospinal fluid could lower anisotropy and skew estimates of diffusion direction in many voxels. We addressed this concern through analyses of global FA, MD, and isotropic diffusion measured within an eroded WM mask, which would be largely independent of partial-volume effects. This analysis again demonstrated greater WM degeneration in non-amnestic than amnestic AD, suggesting that the differences we observed in tractwise analyses could not be attributed entirely to false negatives in tractography.

### The significance of white matter degeneration in Alzheimer’s disease

The present study was motivated by the hypothesis that transneuronal spread of tau would be associated with syndrome-specific patterns of WM degeneration; however, our results do not specify the precise mechanism of degeneration, nor can they definitively rule out alternative explanations. Converging evidence from autopsy studies (Scheltens et al., 1995), neuroimaging (Nasrabady et al., 2018; Veale et al., 2021), and single-cell transcriptomics (Mathys et al., 2019) demonstrates that demyelination is an early and prevalent change in AD that is likely to affect diffusion MRI metrics (Jelescu et al., 2016; Preziosa et al., 2019). Amyloid- β is one possible driver of myelin loss, given prior evidence for toxic effects of soluble amyloid- β on oligodendrocytes (Sachdev et al., 2013; Nasrabady et al., 2018). However, amyloid-related axonal injury is not mutually exclusive with involvement of tau. Agosta et al. (2014) proposed that microvesicles shed by microglia could release neurotoxic oligomeric amyloid- β , which could both cause neuronal and oligodendrocytic damage both directly and indirectly, by promoting tau aggregation and cell injury. Joint involvement of amyloid- β and tau in driving axonal degeneration is also supported by experimental studies of neurons in compartmentalized microfluidic environments, demonstrating that amyloid- β peptide administered to the neuronal soma can induce tau hyperphosphorylation and distal axonal degeneration (Deleglise et al., 2014).

Another possible explanation for the current results is Wallerian degeneration of WM tracts following neuronal injury or death. The cross-sectional design of the current study cannot disambiguate early changes preceding GM degeneration and later changes due to Wallerian processes. However, prior studies have found that WM degeneration precedes GM atrophy in MCI (Agosta et al., 2011; Maier-Hein et al., 2015; Raj et al., 2017) and precedes symptom onset in autosomal-dominant AD by up to 10 years (Araque Caballero et al., 2018). Moreover, Caso et al. (2015) described observed WM degeneration in atypical AD as out of proportion to GM disease, suggesting that WM changes preceded GM atrophy. Ultimately, the distinction between primary WM changes that are instrumental in disease progression vs. subsequent, secondary changes due to Wallerian degeneration may be an artificial one. WM changes such as demyelination and destabilization of microtubules (Kowall and Kosik, 1987) may have cyclical interactions with changes occurring near the neuronal cell body (e.g., tau aggregation, degeneration in somatic structure, nuclear functions, etc.), making it less meaningful to label them as strictly cause or consequence of GM changes. In human observational research, longitudinal imaging is critical to determining the relative sequence of GM vs. WM degeneration; and at the microscopic scale, innovative *in vitro* methods (Deleglise et al., 2014) can help validate mechanistic hypotheses.

### Limitations

The present study has several limitations that urge caution in interpreting our findings. Sample sizes were low for phenotypic contrasts, especially those for bvAD and CBS; although observed WM degeneration was consistent with established patterns of GM atrophy, they require replication with larger datasets. Additionally, we leveraged data acquired from 2007 to 2017 using a single-shell, 30-direction DWI protocol that had limited angular resolution and ability to resolve crossing fibers, which may explain the sparsity and inter-individual variability of structural connectivity matrices. By focusing only on the most reliably-detected fiber tracts, we may in fact have underestimated the extent of WM degeneration across all groups. Additionally, the current analyses are based on a single microstructural metric, generalized fractional anisotropy; but previous studies have demonstrated the sensitivity of alternative metrics like mean diffusivity for detecting WM degeneration in non-amnestic AD (Gatto et al., 2022) and apraxia of speech (Gatto et al., 2024). In future studies, we aim to apply techniques such as neurite orientation dispersion and density imaging (Gatto et al., 2024) to validate the current findings using multi-shell diffusion MRI. One limitation of topological analysis methods as applied in this study is that numbers of connected components and loops are influenced by methodological factors including atlas parcellation; future studies may employ a parcellation-free approach in estimation of topological and graph theoretic metrics (Chung et al., 2011). Finally, we note that biomarker data were unavailable for 14/153 participants (9.2%); these cases are thus characterized as having possible rather than probable AD. While subset analyses omitting these possible AD cases largely replicated the full analysis, the fiber tracts reported in Supplementary Table 5 had discrepant results and should be interpreted with caution.

## Conclusion

The present study demonstrated that non-amnestic AD clinical syndromes were characterized by phenotype-specific patterns of WM degeneration, with greater overall severity than in amnestic AD. The sources of this degeneration are unclear: while the results are broadly consistent with models of transneuronal tau spread, other factors including amyloid- β , ischemia, oxidative stress, and immune responses have all been previously associated with WM disease. Indeed, examining differential involvement of these potential contributors in amnestic vs. non-amnestic AD may be a promising strategy for understanding the group differences in WM disease reported here. The present study also demonstrates that topological metrics—including the number of connected components and closed loops in individuals’ brain graphs—are potential imaging biomarkers of disease state that do not rely on subjective report (as disease duration does) or assessment by human raters (as global cognition does).

## Data availability statement

The raw data supporting the conclusions of this article can be requested through the Penn Neurodegenerative Data Sharing Committee: https://www.pennbindlab.com/data-sharing.

## Ethics statement

The studies involving humans were approved by University of Pennsylvania Institutional Review Board. The studies were conducted in accordance with the local legislation and institutional requirements. Written informed consent for participation in this study was provided by the participants’ legal guardians/next of kin.

## Author contributions

JP: Conceptualization, Data curation, Formal analysis, Funding acquisition, Methodology, Software, Visualization, Writing – original draft, Writing – review & editing. NA: Conceptualization, Formal analysis, Methodology, Software, Validation, Visualization, Writing – original draft, Writing – review & editing. MC: Conceptualization, Investigation, Methodology, Software, Supervision, Writing – review & editing. HR: Data curation, Investigation, Methodology, Software, Validation, Writing – review & editing. CO: Data curation, Investigation, Methodology, Software, Validation, Writing – review & editing. PC: Data curation, Investigation, Methodology, Software, Validation, Writing – review & editing. JG: Funding acquisition, Methodology, Project administration, Resources, Software, Supervision, Writing – review & editing. KC: Formal analysis, Investigation, Validation, Writing – review & editing. SA: Data curation, Validation, Writing - review & editing. DW: Conceptualization, Funding acquisition, Resources, Supervision, Writing – review & editing. CM: Funding acquisition, Resources, Supervision, Writing – review & editing. MG: Conceptualization, Funding acquisition, Investigation, Project administration, Resources, Supervision, Writing – review & editing. DI: Funding acquisition, Project administration, Resources, Writing – review & editing.

## Footnotes

^1^https://github.com/MASILab/Synb0-DISCO

^2^https://dsi-studio.labsolver.org

^3^https://brain.labsolver.org/hcp_trk_atlas.html

^4^https://github.com/jeffrey-phillips
