# Supplementary material for: Greater white matter degeneration and lower structural connectivity in non-amnestic vs. amnestic Alzheimer’s disease
Source: Front Neurosci. 2024 Mar 18;18:1353306. doi: 10.3389/fnins.2024.1353306 (PMC10986184; doi:10.3389/fnins.2024.1353306)
Supplement: Supplementary file 1 [file Table_1.DOCX]

## Supplementary Table 1. Significant between-phenotype differences in neuropsychological performance, based on post-hoc Tukey's tests.

| Task | term | contrast | estimate | std.error | df | statistic | adj.p.value |
| --- | --- | --- | --- | --- | --- | --- | --- |
| Animals | Phenotype | Normal - aAD | 12.057 | 1.567 | 120 | 7.692 | 0.0000 |
|  | Phenotype | Normal - lvPPA | 11.561 | 1.651 | 120 | 7.002 | 0.0000 |
|  | Phenotype | Normal - PCA | 10.677 | 1.942 | 120 | 5.498 | 0.0000 |
|  | Phenotype | Normal - bvAD | 12.607 | 2.344 | 120 | 5.379 | 0.0000 |
|  | Phenotype | Normal - CBS | 12.329 | 2.646 | 120 | 4.659 | 0.0001 |
| Rey figure copy | Phenotype | Normal - aAD | 12.137 | 3.401 | 59 | 3.569 | 0.0090 |
|  | Phenotype | Normal - PCA | 23.626 | 4.038 | 59 | 5.851 | 0.0000 |
|  | Phenotype | Normal - bvAD | 18.184 | 4.614 | 59 | 3.941 | 0.0029 |
|  | Phenotype | Normal - CBS | 26.356 | 7.554 | 59 | 3.489 | 0.0114 |
|  | Phenotype | lvPPA - PCA | 18.179 | 4.021 | 59 | 4.521 | 0.0004 |
| Rey figure recall | Phenotype | Normal - aAD | 15.421 | 2.249 | 59 | 6.858 | 0.0000 |
|  | Phenotype | Normal - PCA | 15.624 | 2.670 | 59 | 5.851 | 0.0000 |
|  | Phenotype | Normal - bvAD | 14.721 | 3.051 | 59 | 4.825 | 0.0001 |
|  | Phenotype | aAD - lvPPA | -10.547 | 2.369 | 59 | -4.452 | 0.0005 |
|  | Phenotype | lvPPA - PCA | 10.750 | 2.659 | 59 | 4.044 | 0.0021 |
|  | Phenotype | lvPPA - bvAD | 9.847 | 3.077 | 59 | 3.200 | 0.0256 |
| Backward digit span | Phenotype | Normal - aAD | 2.394 | 0.339 | 99 | 7.060 | 0.0000 |
|  | Phenotype | Normal - lvPPA | 2.395 | 0.358 | 99 | 6.682 | 0.0000 |
|  | Phenotype | Normal - PCA | 2.493 | 0.421 | 99 | 5.927 | 0.0000 |
|  | Phenotype | Normal - bvAD | 2.372 | 0.483 | 99 | 4.911 | 0.0001 |
|  | Phenotype | Normal - CBS | 3.074 | 0.523 | 99 | 5.880 | 0.0000 |
| Forward digit span | Phenotype | Normal - lvPPA | 2.677 | 0.436 | 99 | 6.144 | 0.0000 |
|  | Phenotype | Normal - PCA | 1.466 | 0.502 | 99 | 2.924 | 0.0477 |
|  | Phenotype | Normal - CBS | 2.426 | 0.636 | 99 | 3.817 | 0.0031 |
|  | Phenotype | aAD - lvPPA | 1.647 | 0.385 | 99 | 4.281 | 0.0006 |
| FAQ | Phenotype | Normal - aAD | -14.323 | 3.842 | 54 | -3.728 | 0.0059 |
|  | Phenotype | Normal - PCA | -15.991 | 4.440 | 54 | -3.601 | 0.0086 |
|  | Phenotype | Normal - bvAD | -23.161 | 5.229 | 54 | -4.429 | 0.0006 |
|  | Phenotype | aAD - lvPPA | 8.829 | 2.480 | 54 | 3.560 | 0.0097 |
|  | Phenotype | lvPPA - PCA | -10.496 | 3.179 | 54 | -3.301 | 0.0201 |
|  | Phenotype | lvPPA - bvAD | -17.666 | 4.293 | 54 | -4.115 | 0.0018 |
| Boston Naming Test | Phenotype | Normal - aAD | 8.510 | 1.802 | 117 | 4.724 | 0.0001 |
|  | Phenotype | Normal - lvPPA | 9.990 | 1.961 | 117 | 5.095 | 0.0000 |
|  | Phenotype | Normal - PCA | 9.045 | 2.244 | 117 | 4.030 | 0.0014 |
|  | Phenotype | Normal - bvAD | 9.315 | 2.699 | 117 | 3.451 | 0.0099 |
|  | Phenotype | Normal - CBS | 13.310 | 3.048 | 117 | 4.367 | 0.0004 |
| NPI-Q | Phenotype | Normal - aAD | -3.611 | 1.085 | 80 | -3.328 | 0.0161 |
|  | Phenotype | Normal - bvAD | -7.073 | 1.340 | 80 | -5.279 | 0.0000 |
|  | Phenotype | aAD - bvAD | -3.463 | 1.040 | 80 | -3.329 | 0.0161 |
|  | Phenotype | lvPPA - bvAD | -4.946 | 1.064 | 80 | -4.647 | 0.0002 |
|  | Phenotype | PCA - bvAD | -4.054 | 1.183 | 80 | -3.425 | 0.0121 |
| F words | Phenotype | Normal - aAD | 7.861 | 1.511 | 99 | 5.203 | 0.0000 |
|  | Phenotype | Normal - lvPPA | 10.150 | 1.580 | 99 | 6.426 | 0.0000 |
|  | Phenotype | Normal - bvAD | 8.262 | 2.155 | 99 | 3.834 | 0.0030 |
|  | Phenotype | Normal - CBS | 10.880 | 2.306 | 99 | 4.717 | 0.0001 |
|  | Phenotype | lvPPA - PCA | -6.027 | 1.671 | 99 | -3.608 | 0.0063 |
| JOLO | Phenotype | Normal - PCA | 3.450 | 0.775 | 75 | 4.451 | 0.0004 |
|  | Phenotype | lvPPA - PCA | 2.945 | 0.680 | 75 | 4.331 | 0.0006 |
|  | Phenotype | PCA - bvAD | -3.051 | 0.942 | 75 | -3.240 | 0.0212 |
| PBAC verbal memory | Phenotype | Normal - aAD | 3.441 | 0.828 | 84 | 4.158 | 0.0011 |
|  | Phenotype | Normal - aAD | 11.518 | 1.513 | 80 | 7.615 | 0.0000 |
|  | Phenotype | Normal - lvPPA | 4.902 | 1.528 | 80 | 3.209 | 0.0227 |
|  | Phenotype | Normal - PCA | 10.609 | 1.674 | 80 | 6.337 | 0.0000 |
|  | Phenotype | Normal - bvAD | 10.034 | 2.043 | 80 | 4.912 | 0.0001 |
|  | Phenotype | Normal - CBS | 6.701 | 2.261 | 80 | 2.964 | 0.0445 |
|  | Phenotype | aAD - lvPPA | -6.616 | 1.194 | 80 | -5.540 | 0.0000 |
|  | Phenotype | lvPPA - PCA | 5.706 | 1.383 | 80 | 4.127 | 0.0012 |

## Supplementary Table 2. Post-hoc tests of group differences in tractwise GFA w-scores.

| Connection ID | term | contrast | estimate | std.error | df | statistic | adj.p.value |
| --- | --- | --- | --- | --- | --- | --- | --- |
| Cerebellum_Region1-Cerebellum_Region3 | Group | naAD - aAD | 0.457 | 0.187 | 142 | 2.451 | 0.0407 |
| LH_Cont_Cing_1-LH_Default_PCC_1 | Group | naAD - aAD | -0.510 | 0.198 | 125 | -2.568 | 0.0304 |
| LH_Cont_Cing_1-LH_Default_PCC_2 | Group | naAD - Normal | -0.553 | 0.199 | 148 | -2.777 | 0.0170 |
| LH_Cont_Cing_1-LH_Default_PCC_2 | Group | Normal - aAD | 0.677 | 0.217 | 148 | 3.115 | 0.0062 |
| LH_Cont_Par_1-LH_Default_Temp_4 | Group | naAD - Normal | -0.967 | 0.251 | 142 | -3.860 | 0.0005 |
| LH_Cont_Par_1-LH_Default_Temp_4 | Group | Normal - aAD | 0.669 | 0.274 | 142 | 2.441 | 0.0418 |
| LH_Cont_pCun_1-LH_Default_PCC_1 | Group | naAD - Normal | -1.093 | 0.306 | 107 | -3.578 | 0.0015 |
| LH_Cont_pCun_1-LH_Default_PCC_1 | Group | Normal - aAD | 0.918 | 0.338 | 107 | 2.719 | 0.0207 |
| LH_Default_PCC_2-RH_Cont_PFCmp_2 | Group | naAD - Normal | -0.796 | 0.244 | 113 | -3.259 | 0.0042 |
| LH_Default_PFC_3-LH_Default_PCC_2 | Group | Normal - aAD | 0.695 | 0.274 | 142 | 2.539 | 0.0325 |
| LH_Default_PFC_3-LH_HTH | Group | Normal - aAD | 1.016 | 0.335 | 83 | 3.036 | 0.0089 |
| LH_Default_PFC_4-LH_Anterior | Group | naAD - aAD | -0.702 | 0.231 | 115 | -3.042 | 0.0081 |
| LH_Default_PFC_4-LH_Pu | Group | Normal - aAD | -0.647 | 0.263 | 149 | -2.457 | 0.0400 |
| LH_Default_Temp_1-LH_Default_Temp_3 | Group | naAD - Normal | -1.690 | 0.450 | 85 | -3.760 | 0.0009 |
| LH_Default_Temp_1-LH_Default_Temp_3 | Group | naAD - aAD | -1.193 | 0.381 | 85 | -3.135 | 0.0066 |
| LH_Default_Temp_2-LH_Default_Temp_3 | Group | naAD - Normal | -0.793 | 0.251 | 133 | -3.152 | 0.0056 |
| LH_Default_Temp_2-LH_Default_Temp_3 | Group | naAD - aAD | -0.838 | 0.216 | 133 | -3.884 | 0.0005 |
| LH_Default_Temp_2-LH_Default_Temp_4 | Group | naAD - Normal | -0.811 | 0.270 | 118 | -3.005 | 0.0090 |
| LH_Default_Temp_3-LH_Default_Temp_4 | Group | naAD - Normal | -0.961 | 0.225 | 146 | -4.268 | 0.0001 |
| LH_Default_Temp_3-LH_Default_Temp_4 | Group | naAD - aAD | -0.479 | 0.195 | 146 | -2.452 | 0.0405 |
| LH_DorsAttn_FEF_1-LH_Default_PFC_6 | Group | naAD - Normal | -0.773 | 0.252 | 131 | -3.073 | 0.0072 |
| LH_DorsAttn_FEF_1-LH_Default_Temp_1 | Group | naAD - aAD | -0.869 | 0.230 | 93 | -3.787 | 0.0008 |
| LH_DorsAttn_FEF_1-LH_Default_Temp_2 | Group | naAD - aAD | -0.600 | 0.207 | 121 | -2.904 | 0.0121 |
| LH_DorsAttn_Post_1-LH_Default_Temp_4 | Group | naAD - Normal | -1.017 | 0.265 | 140 | -3.831 | 0.0006 |
| LH_DorsAttn_Post_1-LH_Default_Temp_4 | Group | naAD - aAD | -0.589 | 0.238 | 140 | -2.474 | 0.0384 |
| LH_DorsAttn_Post_1-LH_Limbic_TempPole_2 | Group | naAD - Normal | -0.493 | 0.194 | 147 | -2.546 | 0.0318 |
| LH_DorsAttn_Post_2-LH_DorsAttn_Post_4 | Group | naAD - Normal | -0.639 | 0.221 | 129 | -2.886 | 0.0126 |
| LH_DorsAttn_Post_2-LH_DorsAttn_Post_4 | Group | Normal - aAD | 0.691 | 0.250 | 129 | 2.764 | 0.0179 |
| LH_DorsAttn_Post_3-LH_Cont_Par_1 | Group | naAD - Normal | -0.580 | 0.203 | 149 | -2.854 | 0.0136 |
| LH_DorsAttn_Post_4-LH_Cont_Par_1 | Group | naAD - Normal | -0.632 | 0.265 | 117 | -2.382 | 0.0490 |
| LH_DorsAttn_Post_6-Cerebellum_Region5 | Group | naAD - Normal | 0.927 | 0.297 | 110 | 3.119 | 0.0065 |
| LH_SalVentAttn_FrOper_1-LH_Default_PFC_1 | Group | naAD - Normal | -0.659 | 0.248 | 139 | -2.656 | 0.0239 |
| LH_SalVentAttn_Med_1-LH_Default_PCC_2 | Group | naAD - Normal | -0.741 | 0.232 | 149 | -3.193 | 0.0049 |
| LH_SalVentAttn_Med_1-LH_Default_PFC_3 | Group | naAD - Normal | -0.753 | 0.245 | 142 | -3.074 | 0.0071 |
| LH_SalVentAttn_Med_3-RH_SomMot_8 | Group | naAD - Normal | -1.451 | 0.413 | 109 | -3.516 | 0.0018 |
| LH_SalVentAttn_Med_3-RH_SomMot_8 | Group | Normal - aAD | 1.039 | 0.423 | 109 | 2.456 | 0.0411 |
| LH_SalVentAttn_PFCl_1-LH_Default_PFC_5 | Group | naAD - Normal | -0.572 | 0.209 | 142 | -2.732 | 0.0193 |
| LH_SalVentAttn_PFCl_1-LH_Default_PFC_7 | Group | naAD - Normal | -0.834 | 0.245 | 128 | -3.405 | 0.0025 |
| LH_SomMot_1-LH_Default_Temp_2 | Group | naAD - aAD | -0.573 | 0.223 | 119 | -2.573 | 0.0302 |
| LH_SomMot_1-LH_Default_Temp_2 | Group | Normal - aAD | -0.849 | 0.284 | 119 | -2.993 | 0.0093 |
| LH_SomMot_2-LH_SalVentAttn_FrOper_1 | Group | naAD - Normal | -0.650 | 0.212 | 141 | -3.063 | 0.0074 |
| LH_SomMot_4-LH_DorsAttn_Post_4 | Group | naAD - Normal | -0.650 | 0.239 | 101 | -2.714 | 0.0212 |
| LH_SomMot_4-LH_SomMot_5 | Group | naAD - Normal | -0.548 | 0.228 | 139 | -2.405 | 0.0458 |
| LH_SomMot_5-LH_Cont_Par_1 | Group | Normal - aAD | 1.035 | 0.364 | 98 | 2.844 | 0.0149 |
| LH_SomMot_6-Cerebellum_Region5 | Group | naAD - Normal | 0.598 | 0.240 | 138 | 2.496 | 0.0363 |
| LH_SomMot_6-LH_Default_PCC_2 | Group | naAD - Normal | -0.800 | 0.300 | 89 | -2.667 | 0.0244 |
| LH_SomMot_6-LH_DorsAttn_Post_5 | Group | naAD - Normal | -1.108 | 0.305 | 86 | -3.638 | 0.0013 |
| LH_SomMot_6-LH_SalVentAttn_Med_2 | Group | naAD - Normal | -0.998 | 0.272 | 127 | -3.675 | 0.0010 |
| LH_SomMot_6-LH_SalVentAttn_Med_2 | Group | Normal - aAD | 0.997 | 0.293 | 127 | 3.406 | 0.0025 |
| LH_SomMot_6-RH_SomMot_8 | Group | naAD - Normal | -0.818 | 0.327 | 140 | -2.499 | 0.0361 |
| LH_Vis_1-LH_Default_PCC_1 | Group | naAD - Normal | -0.565 | 0.223 | 102 | -2.531 | 0.0342 |
| LH_Vis_2-LH_Limbic_TempPole_2 | Group | naAD - Normal | -0.840 | 0.236 | 86 | -3.561 | 0.0017 |
| LH_Vis_2-LH_Limbic_TempPole_2 | Group | Normal - aAD | 0.720 | 0.250 | 86 | 2.877 | 0.0139 |
| LH_Vis_4-LH_Vis_8 | Group | Normal - aAD | 0.600 | 0.240 | 144 | 2.503 | 0.0356 |
| LH_Vis_7-LH_Default_Temp_4 | Group | naAD - Normal | -0.674 | 0.210 | 141 | -3.207 | 0.0047 |
| LH_Vis_7-LH_DorsAttn_Post_1 | Group | naAD - Normal | -1.140 | 0.235 | 143 | -4.852 | 0.0000 |
| LH_Vis_7-LH_DorsAttn_Post_1 | Group | naAD - aAD | -0.581 | 0.206 | 143 | -2.815 | 0.0153 |
| LH_Vis_7-LH_Vis_8 | Group | naAD - Normal | -0.672 | 0.183 | 139 | -3.676 | 0.0010 |
| LH_Vis_8-LH_DorsAttn_Post_1 | Group | naAD - Normal | -0.602 | 0.194 | 138 | -3.096 | 0.0067 |
| RH_Cont_PFCl_1-RH_Default_PFCm_2 | Group | naAD - aAD | -0.659 | 0.224 | 112 | -2.939 | 0.0111 |
| RH_Cont_PFCl_2-RH_Default_PFCm_2 | Group | naAD - Normal | -0.725 | 0.270 | 117 | -2.686 | 0.0224 |
| RH_Cont_PFCmp_1-RH_Cont_PFCmp_2 | Group | Normal - aAD | 0.693 | 0.251 | 140 | 2.755 | 0.0182 |
| RH_Cont_PFCmp_1-RH_Cont_PFCmp_3 | Group | naAD - Normal | -0.634 | 0.245 | 119 | -2.589 | 0.0290 |
| RH_Cont_PFCmp_1-RH_Default_PCC_2 | Group | naAD - Normal | -0.943 | 0.278 | 148 | -3.388 | 0.0026 |
| RH_Cont_PFCmp_1-RH_Default_PFCm_1 | Group | naAD - Normal | -0.958 | 0.346 | 93 | -2.769 | 0.0184 |
| RH_Cont_PFCmp_2-RH_Cont_PFCmp_3 | Group | naAD - Normal | -0.616 | 0.240 | 120 | -2.568 | 0.0306 |
| RH_Cont_PFCmp_2-RH_Default_PCC_2 | Group | naAD - Normal | -0.820 | 0.219 | 148 | -3.743 | 0.0008 |
| RH_Cont_PFCmp_2-RH_Default_PCC_2 | Group | Normal - aAD | 0.704 | 0.238 | 148 | 2.954 | 0.0102 |
| RH_Cont_PFCmp_2-RH_Default_PFCm_1 | Group | Normal - aAD | 0.866 | 0.310 | 135 | 2.795 | 0.0163 |
| RH_Default_PFCm_1-RH_Default_PCC_2 | Group | naAD - Normal | -0.675 | 0.228 | 134 | -2.959 | 0.0101 |
| RH_Default_PFCm_1-RH_Default_PCC_2 | Group | Normal - aAD | 0.807 | 0.249 | 134 | 3.236 | 0.0043 |
| RH_Default_Temp_1-RH_Default_Temp_3 | Group | naAD - aAD | -0.809 | 0.330 | 114 | -2.447 | 0.0418 |
| RH_DorsAttn_FEF_1-RH_Cont_Par_2 | Group | naAD - aAD | -0.712 | 0.234 | 127 | -3.045 | 0.0079 |
| RH_DorsAttn_Post_1-RH_Cont_Par_2 | Group | naAD - Normal | -0.997 | 0.269 | 131 | -3.702 | 0.0009 |
| RH_DorsAttn_Post_1-RH_Cont_Par_2 | Group | Normal - aAD | 0.828 | 0.290 | 131 | 2.859 | 0.0136 |
| RH_DorsAttn_Post_1-RH_Default_Par_1 | Group | naAD - Normal | -0.883 | 0.289 | 134 | -3.057 | 0.0076 |
| RH_DorsAttn_Post_1-RH_Default_Par_1 | Group | naAD - aAD | -0.636 | 0.260 | 134 | -2.449 | 0.0411 |
| RH_DorsAttn_Post_1-RH_Default_Temp_1 | Group | naAD - Normal | -1.251 | 0.299 | 115 | -4.187 | 0.0002 |
| RH_DorsAttn_Post_1-RH_Default_Temp_1 | Group | Normal - aAD | 0.810 | 0.317 | 115 | 2.558 | 0.0315 |
| RH_DorsAttn_Post_1-RH_SalVentAttn_TempOccPar_1 | Group | naAD - Normal | -0.568 | 0.230 | 107 | -2.468 | 0.0399 |
| RH_DorsAttn_Post_3-RH_Cont_Par_2 | Group | naAD - Normal | -0.576 | 0.234 | 139 | -2.462 | 0.0397 |
| RH_DorsAttn_Post_4-RH_Cont_Par_2 | Group | naAD - Normal | -0.986 | 0.283 | 144 | -3.491 | 0.0018 |
| RH_DorsAttn_Post_4-RH_Cont_Par_2 | Group | Normal - aAD | 0.862 | 0.311 | 144 | 2.777 | 0.0170 |
| RH_Limbic_OFC_1-RH_Ca | Group | naAD - aAD | -0.581 | 0.218 | 135 | -2.667 | 0.0232 |
| RH_Limbic_TempPole_1-RH_Ca | Group | naAD - Normal | -0.565 | 0.223 | 120 | -2.539 | 0.0330 |
| RH_Limbic_TempPole_1-RH_NAC | Group | naAD - Normal | -0.625 | 0.229 | 120 | -2.728 | 0.0199 |
| RH_SalVentAttn_Med_1-RH_Cont_PFCmp_2 | Group | naAD - Normal | -0.888 | 0.263 | 125 | -3.374 | 0.0028 |
| RH_SalVentAttn_Med_1-RH_SalVentAttn_Med_2 | Group | naAD - Normal | -0.741 | 0.305 | 140 | -2.432 | 0.0428 |
| RH_SalVentAttn_Med_2-RH_Cont_PFCmp_2 | Group | naAD - Normal | -0.822 | 0.305 | 135 | -2.691 | 0.0218 |
| RH_SalVentAttn_Med_2-RH_Cont_PFCmp_2 | Group | naAD - aAD | -0.881 | 0.264 | 135 | -3.332 | 0.0032 |
| RH_SalVentAttn_Med_2-RH_Pu | Group | Normal - aAD | -0.608 | 0.251 | 123 | -2.425 | 0.0439 |
| RH_SalVentAttn_TempOccPar_1-RH_Default_Par_1 | Group | naAD - Normal | -0.703 | 0.278 | 133 | -2.533 | 0.0332 |
| RH_SalVentAttn_TempOccPar_1-RH_Default_Temp_3 | Group | naAD - Normal | -0.961 | 0.280 | 127 | -3.435 | 0.0023 |
| RH_SomMot_1-RH_SalVentAttn_TempOccPar_2 | Group | naAD - aAD | -0.387 | 0.158 | 134 | -2.453 | 0.0407 |
| RH_SomMot_5-RH_SomMot_6 | Group | Normal - aAD | 0.653 | 0.249 | 136 | 2.619 | 0.0264 |
| RH_SomMot_8-RH_DorsAttn_FEF_2 | Group | naAD - Normal | -0.836 | 0.263 | 108 | -3.173 | 0.0055 |
| RH_Vis_1-RH_Default_PCC_1 | Group | naAD - Normal | -0.622 | 0.224 | 114 | -2.780 | 0.0173 |
| RH_Vis_1-RH_Default_PCC_1 | Group | Normal - aAD | 0.805 | 0.256 | 114 | 3.148 | 0.0059 |
| RH_Vis_2-RH_Vis_8 | Group | naAD - Normal | -0.620 | 0.252 | 121 | -2.462 | 0.0401 |
| RH_Vis_3-RH_DorsAttn_Post_1 | Group | naAD - Normal | -0.872 | 0.248 | 149 | -3.522 | 0.0016 |
| RH_Vis_3-RH_DorsAttn_Post_1 | Group | Normal - aAD | 0.732 | 0.270 | 149 | 2.705 | 0.0207 |
| RH_Vis_3-RH_Vis_7 | Group | naAD - Normal | -0.814 | 0.241 | 145 | -3.376 | 0.0027 |
| RH_Vis_3-RH_Vis_8 | Group | naAD - Normal | -0.626 | 0.232 | 111 | -2.693 | 0.0221 |
| RH_Vis_4-RH_Vis_7 | Group | naAD - Normal | -0.591 | 0.226 | 129 | -2.610 | 0.0272 |
| RH_Vis_7-RH_Vis_8 | Group | naAD - Normal | -0.588 | 0.226 | 142 | -2.603 | 0.0274 |
| RH_Vis_7-RH_Vis_8 | Group | Normal - aAD | 0.617 | 0.253 | 142 | 2.437 | 0.0422 |

## Supplementary Table 3. Post-hoc tests of phenotypic differences in tractwise GFA w-scores.

| Connection ID | term | contrast | estimate | std.error | df | statistic | adj.p.value |
| --- | --- | --- | --- | --- | --- | --- | --- |
| Cerebellum_Region2-Cerebellum_Region5 | Phenotype | Normal - CBS | -1.969 | 0.486 | 135 | -4.051 | 0.0012 |
| Cerebellum_Region2-Cerebellum_Region5 | Phenotype | aAD - CBS | -1.750 | 0.454 | 135 | -3.854 | 0.0024 |
| Cerebellum_Region2-Cerebellum_Region5 | Phenotype | lvPPA - CBS | -1.867 | 0.462 | 135 | -4.040 | 0.0012 |
| Cerebellum_Region2-Cerebellum_Region5 | Phenotype | PCA - CBS | -1.556 | 0.511 | 135 | -3.048 | 0.0324 |
| LH_Cont_Cing_1-LH_Default_PCC_2 | Phenotype | Normal - aAD | 0.672 | 0.220 | 145 | 3.061 | 0.0309 |
| LH_Cont_Par_1-LH_Default_Temp_4 | Phenotype | Normal - PCA | 1.283 | 0.329 | 139 | 3.903 | 0.0020 |
| LH_Cont_pCun_1-LH_Default_PCC_1 | Phenotype | Normal - lvPPA | 1.169 | 0.352 | 104 | 3.322 | 0.0152 |
| LH_Cont_pCun_1-LH_Default_Temp_1 | Phenotype | bvAD - CBS | -1.673 | 0.532 | 92 | -3.147 | 0.0262 |
| LH_Default_PCC_2-RH_Cont_PFCmp_2 | Phenotype | Normal - PCA | 0.929 | 0.317 | 110 | 2.934 | 0.0457 |
| LH_Default_PCC_2-RH_Cont_PFCmp_2 | Phenotype | Normal - bvAD | 1.341 | 0.409 | 110 | 3.282 | 0.0169 |
| LH_Default_PFC_3-RH_Limbic_OFC_1 | Phenotype | Normal - bvAD | 1.459 | 0.447 | 137 | 3.267 | 0.0169 |
| LH_Default_PFC_3-RH_Limbic_OFC_1 | Phenotype | bvAD - CBS | -1.810 | 0.550 | 137 | -3.291 | 0.0157 |
| LH_Default_PFC_4-LH_Anterior | Phenotype | aAD - lvPPA | 0.861 | 0.282 | 112 | 3.059 | 0.0323 |
| LH_Default_Temp_1-LH_Default_Temp_3 | Phenotype | Normal - lvPPA | 1.659 | 0.518 | 82 | 3.203 | 0.0230 |
| LH_Default_Temp_1-LH_Default_Temp_3 | Phenotype | Normal - bvAD | 2.463 | 0.779 | 82 | 3.164 | 0.0257 |
| LH_Default_Temp_2-LH_Default_Temp_4 | Phenotype | Normal - PCA | 1.240 | 0.372 | 115 | 3.333 | 0.0143 |
| LH_Default_Temp_3-LH_Default_Temp_4 | Phenotype | Normal - lvPPA | 0.906 | 0.259 | 143 | 3.503 | 0.0079 |
| LH_Default_Temp_3-LH_Default_Temp_4 | Phenotype | Normal - PCA | 1.032 | 0.296 | 143 | 3.483 | 0.0085 |
| LH_Default_Temp_3-LH_Default_Temp_4 | Phenotype | Normal - CBS | 1.344 | 0.408 | 143 | 3.289 | 0.0157 |
| LH_DorsAttn_FEF_1-LH_Default_Temp_1 | Phenotype | aAD - lvPPA | 0.912 | 0.263 | 90 | 3.461 | 0.0104 |
| LH_DorsAttn_FEF_1-LH_Default_Temp_1 | Phenotype | aAD - bvAD | 1.166 | 0.382 | 90 | 3.052 | 0.0344 |
| LH_DorsAttn_Post_1-LH_Default_Temp_4 | Phenotype | Normal - lvPPA | 0.915 | 0.302 | 137 | 3.034 | 0.0336 |
| LH_DorsAttn_Post_1-LH_Default_Temp_4 | Phenotype | Normal - PCA | 1.166 | 0.360 | 137 | 3.242 | 0.0183 |
| LH_DorsAttn_Post_1-LH_Default_Temp_4 | Phenotype | Normal - CBS | 1.797 | 0.500 | 137 | 3.594 | 0.0059 |
| LH_DorsAttn_Post_2-LH_DorsAttn_Post_4 | Phenotype | Normal - bvAD | 1.122 | 0.352 | 126 | 3.187 | 0.0219 |
| LH_Limbic_OFC_1-RH_Default_PFCm_1 | Phenotype | Normal - bvAD | 1.530 | 0.427 | 126 | 3.580 | 0.0064 |
| LH_Limbic_OFC_1-RH_Default_PFCm_1 | Phenotype | bvAD - CBS | -1.766 | 0.523 | 126 | -3.373 | 0.0124 |
| LH_Limbic_OFC_1-RH_Limbic_OFC_1 | Phenotype | Normal - bvAD | 1.187 | 0.403 | 137 | 2.948 | 0.0428 |
| LH_Limbic_TempPole_1-LH_Ca | Phenotype | Normal - bvAD | 1.356 | 0.432 | 113 | 3.138 | 0.0258 |
| LH_RN-Cerebellum_Region5 | Phenotype | PCA - bvAD | 1.276 | 0.395 | 110 | 3.235 | 0.0195 |
| LH_SalVentAttn_Med_1-LH_Default_PCC_2 | Phenotype | Normal - PCA | 1.001 | 0.305 | 146 | 3.281 | 0.0160 |
| LH_SalVentAttn_Med_1-LH_Default_PCC_2 | Phenotype | Normal - bvAD | 1.274 | 0.386 | 146 | 3.300 | 0.0151 |
| LH_SalVentAttn_Med_2-RH_SomMot_8 | Phenotype | lvPPA - CBS | 1.824 | 0.611 | 86 | 2.985 | 0.0415 |
| LH_SalVentAttn_Med_3-RH_SalVentAttn_Med_2 | Phenotype | Normal - CBS | 2.178 | 0.647 | 122 | 3.367 | 0.0127 |
| LH_SalVentAttn_Med_3-RH_SomMot_8 | Phenotype | Normal - bvAD | 2.909 | 0.644 | 106 | 4.514 | 0.0002 |
| LH_SalVentAttn_Med_3-RH_SomMot_8 | Phenotype | Normal - CBS | 3.770 | 1.055 | 106 | 3.572 | 0.0069 |
| LH_SalVentAttn_Med_3-RH_SomMot_8 | Phenotype | aAD - bvAD | 1.889 | 0.603 | 106 | 3.131 | 0.0266 |
| LH_SalVentAttn_Med_3-RH_SomMot_8 | Phenotype | lvPPA - bvAD | 2.020 | 0.619 | 106 | 3.264 | 0.0180 |
| LH_SalVentAttn_PFCl_1-LH_Default_PFC_5 | Phenotype | Normal - PCA | 0.842 | 0.272 | 139 | 3.091 | 0.0285 |
| LH_SalVentAttn_PFCl_1-LH_Default_PFC_7 | Phenotype | Normal - PCA | 0.938 | 0.320 | 125 | 2.934 | 0.0450 |
| LH_SalVentAttn_PFCl_1-LH_Default_PFC_7 | Phenotype | Normal - bvAD | 1.178 | 0.402 | 125 | 2.930 | 0.0454 |
| LH_SomMot_1-LH_Default_Temp_2 | Phenotype | Normal - aAD | -0.838 | 0.288 | 116 | -2.913 | 0.0480 |
| LH_SomMot_6-LH_Default_PCC_2 | Phenotype | Normal - PCA | 1.189 | 0.393 | 86 | 3.027 | 0.0371 |
| LH_SomMot_6-LH_DorsAttn_Post_5 | Phenotype | Normal - PCA | 1.263 | 0.403 | 83 | 3.138 | 0.0275 |
| LH_SomMot_6-LH_DorsAttn_Post_5 | Phenotype | Normal - CBS | 1.827 | 0.580 | 83 | 3.151 | 0.0266 |
| LH_SomMot_6-LH_SalVentAttn_Med_2 | Phenotype | Normal - aAD | 1.017 | 0.295 | 124 | 3.449 | 0.0098 |
| LH_SomMot_6-LH_SalVentAttn_Med_2 | Phenotype | Normal - lvPPA | 0.981 | 0.315 | 124 | 3.117 | 0.0270 |
| LH_SomMot_6-LH_SalVentAttn_Med_2 | Phenotype | Normal - CBS | 1.508 | 0.466 | 124 | 3.232 | 0.0192 |
| LH_SomMot_6-RH_SomMot_8 | Phenotype | Normal - bvAD | 1.612 | 0.534 | 137 | 3.020 | 0.0350 |
| LH_Vis_2-LH_Limbic_TempPole_2 | Phenotype | Normal - lvPPA | 0.900 | 0.267 | 83 | 3.379 | 0.0137 |
| LH_Vis_5-RH_Vis_6 | Phenotype | Normal - lvPPA | 1.205 | 0.349 | 88 | 3.456 | 0.0106 |
| LH_Vis_5-RH_Vis_6 | Phenotype | Normal - bvAD | 2.514 | 0.501 | 88 | 5.019 | 0.0000 |
| LH_Vis_5-RH_Vis_6 | Phenotype | aAD - bvAD | 2.054 | 0.455 | 88 | 4.517 | 0.0003 |
| LH_Vis_5-RH_Vis_6 | Phenotype | lvPPA - PCA | -1.171 | 0.396 | 88 | -2.953 | 0.0451 |
| LH_Vis_5-RH_Vis_6 | Phenotype | lvPPA - CBS | -1.732 | 0.483 | 88 | -3.588 | 0.0070 |
| LH_Vis_5-RH_Vis_6 | Phenotype | PCA - bvAD | 2.480 | 0.511 | 88 | 4.858 | 0.0001 |
| LH_Vis_5-RH_Vis_6 | Phenotype | bvAD - CBS | -3.042 | 0.562 | 88 | -5.411 | 0.0000 |
| LH_Vis_5-RH_Vis_8 | Phenotype | bvAD - CBS | -1.791 | 0.498 | 126 | -3.596 | 0.0060 |
| LH_Vis_7-LH_Default_Temp_4 | Phenotype | Normal - CBS | 1.219 | 0.395 | 138 | 3.085 | 0.0291 |
| LH_Vis_7-LH_DorsAttn_Post_1 | Phenotype | Normal - lvPPA | 1.132 | 0.270 | 140 | 4.195 | 0.0007 |
| LH_Vis_7-LH_DorsAttn_Post_1 | Phenotype | Normal - PCA | 1.340 | 0.320 | 140 | 4.188 | 0.0007 |
| LH_Vis_7-LH_Vis_8 | Phenotype | Normal - PCA | 0.790 | 0.237 | 136 | 3.337 | 0.0137 |
| LH_Vis_8-RH_Vis_8 | Phenotype | aAD - bvAD | 1.855 | 0.536 | 108 | 3.463 | 0.0097 |
| LH_Vis_9-RH_Vis_6 | Phenotype | Normal - bvAD | 1.354 | 0.422 | 138 | 3.206 | 0.0203 |
| RH_Cont_PFCmp_1-RH_Default_PCC_2 | Phenotype | Normal - bvAD | 1.876 | 0.480 | 145 | 3.904 | 0.0020 |
| RH_Cont_PFCmp_2-RH_Default_PCC_2 | Phenotype | Normal - aAD | 0.727 | 0.239 | 145 | 3.043 | 0.0326 |
| RH_Cont_PFCmp_2-RH_Default_PCC_2 | Phenotype | Normal - lvPPA | 0.731 | 0.248 | 145 | 2.942 | 0.0432 |
| RH_Cont_PFCmp_2-RH_Default_PCC_2 | Phenotype | Normal - bvAD | 1.377 | 0.366 | 145 | 3.760 | 0.0033 |
| RH_Default_PFCm_1-RH_Default_PCC_2 | Phenotype | Normal - aAD | 0.816 | 0.252 | 131 | 3.232 | 0.0190 |
| RH_DorsAttn_Post_1-RH_Cont_Par_2 | Phenotype | Normal - aAD | 0.830 | 0.282 | 128 | 2.938 | 0.0443 |
| RH_DorsAttn_Post_1-RH_Cont_Par_2 | Phenotype | Normal - PCA | 1.552 | 0.356 | 128 | 4.356 | 0.0004 |
| RH_DorsAttn_Post_1-RH_Cont_Par_2 | Phenotype | Normal - CBS | 1.805 | 0.509 | 128 | 3.548 | 0.0070 |
| RH_DorsAttn_Post_1-RH_Default_Par_1 | Phenotype | Normal - PCA | 1.109 | 0.380 | 131 | 2.914 | 0.0472 |
| RH_DorsAttn_Post_1-RH_Default_Par_1 | Phenotype | Normal - CBS | 1.899 | 0.540 | 131 | 3.517 | 0.0077 |
| RH_DorsAttn_Post_1-RH_Default_Par_1 | Phenotype | aAD - CBS | 1.590 | 0.503 | 131 | 3.158 | 0.0237 |
| RH_DorsAttn_Post_1-RH_Default_Temp_1 | Phenotype | Normal - PCA | 1.844 | 0.401 | 112 | 4.594 | 0.0002 |
| RH_DorsAttn_Post_1-RH_Default_Temp_1 | Phenotype | Normal - bvAD | 1.514 | 0.458 | 112 | 3.307 | 0.0157 |
| RH_DorsAttn_Post_1-RH_SalVentAttn_TempOccPar_1 | Phenotype | Normal - PCA | 1.001 | 0.284 | 104 | 3.529 | 0.0080 |
| RH_DorsAttn_Post_1-RH_SalVentAttn_TempOccPar_1 | Phenotype | aAD - PCA | 0.851 | 0.270 | 104 | 3.157 | 0.0248 |
| RH_DorsAttn_Post_4-RH_Cont_Par_2 | Phenotype | Normal - aAD | 0.908 | 0.308 | 141 | 2.950 | 0.0424 |
| RH_DorsAttn_Post_4-RH_Cont_Par_2 | Phenotype | Normal - PCA | 1.106 | 0.368 | 141 | 3.010 | 0.0359 |
| RH_DorsAttn_Post_4-RH_Cont_Par_2 | Phenotype | Normal - bvAD | 1.454 | 0.464 | 141 | 3.133 | 0.0252 |
| RH_DorsAttn_Post_4-RH_Cont_Par_2 | Phenotype | Normal - CBS | 1.825 | 0.531 | 141 | 3.435 | 0.0099 |
| RH_Limbic_OFC_1-RH_Ca | Phenotype | aAD - bvAD | 1.122 | 0.375 | 132 | 2.991 | 0.0382 |
| RH_SalVentAttn_FrOper_1-RH_Default_Temp_2 | Phenotype | Normal - PCA | 1.008 | 0.324 | 134 | 3.111 | 0.0271 |
| RH_SalVentAttn_Med_1-RH_Cont_PFCmp_2 | Phenotype | Normal - PCA | 1.090 | 0.366 | 122 | 2.977 | 0.0401 |
| RH_SalVentAttn_Med_1-RH_SalVentAttn_Med_2 | Phenotype | Normal - PCA | 1.438 | 0.397 | 137 | 3.621 | 0.0054 |
| RH_SalVentAttn_Med_1-RH_SalVentAttn_Med_2 | Phenotype | lvPPA - PCA | 1.209 | 0.391 | 137 | 3.091 | 0.0285 |
| RH_SalVentAttn_Med_2-RH_Cont_PFCmp_2 | Phenotype | aAD - PCA | 1.155 | 0.394 | 132 | 2.935 | 0.0445 |
| RH_SalVentAttn_Med_2-RH_Ventral_Latero_Ventral | Phenotype | Normal - CBS | -1.829 | 0.517 | 81 | -3.536 | 0.0086 |
| RH_SalVentAttn_TempOccPar_1-RH_Default_Par_1 | Phenotype | Normal - PCA | 1.216 | 0.390 | 130 | 3.116 | 0.0268 |
| RH_SalVentAttn_TempOccPar_1-RH_Default_Temp_1 | Phenotype | Normal - PCA | 1.231 | 0.420 | 89 | 2.930 | 0.0478 |
| RH_SalVentAttn_TempOccPar_1-RH_Default_Temp_3 | Phenotype | Normal - PCA | 1.846 | 0.374 | 124 | 4.942 | 0.0000 |
| RH_SalVentAttn_TempOccPar_1-RH_Default_Temp_3 | Phenotype | Normal - CBS | 1.572 | 0.490 | 124 | 3.211 | 0.0204 |
| RH_SalVentAttn_TempOccPar_1-RH_Default_Temp_3 | Phenotype | aAD - PCA | 1.318 | 0.376 | 124 | 3.508 | 0.0081 |
| RH_SalVentAttn_TempOccPar_1-RH_Default_Temp_3 | Phenotype | lvPPA - PCA | 1.462 | 0.381 | 124 | 3.834 | 0.0027 |
| RH_SomMot_3-RH_SalVentAttn_FrOper_1 | Phenotype | PCA - bvAD | 1.590 | 0.524 | 104 | 3.034 | 0.0351 |
| RH_SomMot_4-RH_SomMot_6 | Phenotype | Normal - bvAD | 1.760 | 0.489 | 106 | 3.603 | 0.0062 |
| RH_SomMot_4-RH_SomMot_6 | Phenotype | lvPPA - bvAD | 1.471 | 0.465 | 106 | 3.163 | 0.0243 |
| RH_SomMot_8-Cerebellum_Region2 | Phenotype | lvPPA - CBS | -1.381 | 0.464 | 134 | -2.979 | 0.0393 |
| RH_Vis_1-RH_Default_PCC_1 | Phenotype | Normal - aAD | 0.819 | 0.255 | 111 | 3.215 | 0.0207 |
| RH_Vis_1-RH_Default_PCC_1 | Phenotype | Normal - bvAD | 1.150 | 0.375 | 111 | 3.063 | 0.0320 |
| RH_Vis_2-RH_Vis_8 | Phenotype | Normal - PCA | 1.047 | 0.330 | 118 | 3.175 | 0.0229 |
| RH_Vis_3-RH_DorsAttn_Post_1 | Phenotype | Normal - PCA | 1.575 | 0.314 | 146 | 5.018 | 0.0000 |
| RH_Vis_3-RH_DorsAttn_Post_1 | Phenotype | lvPPA - PCA | 1.238 | 0.314 | 146 | 3.941 | 0.0017 |
| RH_Vis_3-RH_Vis_7 | Phenotype | Normal - PCA | 1.287 | 0.316 | 142 | 4.075 | 0.0011 |
| RH_Vis_3-RH_Vis_8 | Phenotype | Normal - PCA | 0.881 | 0.298 | 108 | 2.954 | 0.0434 |
| RH_Vis_7-RH_Vis_8 | Phenotype | Normal - PCA | 1.206 | 0.291 | 139 | 4.149 | 0.0008 |
| RH_Vis_7-RH_Vis_8 | Phenotype | lvPPA - PCA | 0.901 | 0.289 | 139 | 3.112 | 0.0268 |

## Supplementary Table 4. Correlation of Betti-0 and Betti-1 numbers (based on untransformed and w-score-normalized graphs) with clinical and diffusion microstructure variables.

| Variable | GFA_B0 | GFA_B0_p | GFA_B1 | GFA_B1_p | GFA_Bratio | GFA_Bratio_p | W-score_B0 | W-score_B0_p | W-score_B1 | W-score_B1_p | W-score_Bratio | W-score_Bratio_p |
| --- | --- | --- | --- | --- | --- | --- | --- | --- | --- | --- | --- | --- |
| Age | -0.03123511 | 1.000000000 | -0.065843094 | 1.00000000 | -0.013422828 | 1.000000000 | -0.08617736 | 1.000000000 | 0.03717454 | 1.00000000 | 0.07346485 | 1.00000000 |
| SexMale | -0.02256748 | 1.000000000 | -0.002725962 | 1.00000000 | 0.006419027 | 1.000000000 | -0.16731621 | 0.851714867 | 0.13884704 | 1.00000000 | 0.16597367 | 0.85171487 |
| Duration | 0.27970122 | 0.016655687 | -0.281334910 | 0.01579891 | -0.293140533 | 0.009191718 | 0.30769712 | 0.004369741 | -0.23798251 | 0.08121518 | -0.26054922 | 0.03657333 |
| MMSETotal | -0.29019705 | 0.010411661 | 0.248099954 | 0.05762589 | 0.259009028 | 0.037995276 | -0.23922006 | 0.081215181 | 0.15047236 | 1.00000000 | 0.20199401 | 0.28251233 |
| fa | -0.35466806 | 0.000308888 | 0.278298487 | 0.01810698 | 0.209044709 | 0.233965866 | -0.07265092 | 1.000000000 | 0.04873212 | 1.00000000 | 0.02245815 | 1.00000000 |
| md | -0.25975745 | 0.037995276 | 0.276296310 | 0.01937199 | 0.312163101 | 0.003704580 | 0.08493374 | 1.000000000 | -0.03492186 | 1.00000000 | -0.06717731 | 1.00000000 |
| iso | 0.23097709 | 0.104916636 | -0.268040516 | 0.02778115 | -0.239821604 | 0.081215181 | 0.09936256 | 1.000000000 | -0.05623797 | 1.00000000 | -0.07714498 | 1.00000000 |

## Supplementary Table 5. Fiber tracts with differing statistical significance for main effects of group or phenotype in full (n=153) vs. subset (n=139) analyses.

| Connection ID | term | sumsq | meansq | statistic | p.value | FDR | sumsq_n139 | meansq_n139 | statistic_n139 | p.value_n139 | FDR_n139 |
| --- | --- | --- | --- | --- | --- | --- | --- | --- | --- | --- | --- |
| LH_DorsAttn_FEF_1-LH_Default_Temp_1 | Group | 12.50 | 6.249 | 6.99 | 0.00148 | 0.00148 | 5.36 | 2.678 | 2.93 | 0.05904 | 0.05904 |
| LH_DorsAttn_FEF_1-LH_Default_Temp_1 | Phenotype | 16.61 | 3.321 | 3.79 | 0.00371 | 0.00371 | 9.53 | 1.906 | 2.12 | 0.07071 | 0.07071 |
| LH_DorsAttn_Post_3-LH_Cont_pCun_1 | Group | 8.14 | 4.071 | 5.30 | 0.00625 | 0.00625 | 4.64 | 2.321 | 2.95 | 0.05669 | 0.05669 |
| LH_DorsAttn_FEF_1-LH_Default_Temp_2 | Group | 8.24 | 4.120 | 4.75 | 0.01029 | 0.01029 | 5.01 | 2.503 | 2.75 | 0.06864 | 0.06864 |
| RH_DorsAttn_FEF_1-RH_Cont_Par_2 | Group | 10.90 | 5.450 | 4.66 | 0.01119 | 0.01119 | 5.12 | 2.558 | 2.35 | 0.10029 | 0.10029 |
| LH_SalVentAttn_Med_3-LH_Medio_Dorsal | Group | 9.74 | 4.871 | 4.41 | 0.01407 | 0.01407 | 6.25 | 3.125 | 2.87 | 0.06065 | 0.06065 |
| RH_Ca-RH_Ventral_Latero_Dorsal | Group | 8.44 | 4.218 | 4.10 | 0.01888 | 0.01888 | 4.92 | 2.458 | 2.43 | 0.09298 | 0.09298 |
| LH_Cont_Cing_1-LH_Default_PCC_1 | Group | 6.69 | 3.343 | 4.09 | 0.01895 | 0.01895 | 4.34 | 2.171 | 2.54 | 0.08354 | 0.08354 |
| RH_SomMot_5-RH_SomMot_6 | Phenotype | 12.01 | 2.401 | 2.71 | 0.02281 | 0.02281 | 9.86 | 1.971 | 2.24 | 0.05495 | 0.05495 |
| LH_Default_PFC_3-LH_HTH | Phenotype | 13.85 | 2.770 | 2.69 | 0.02659 | 0.02659 | 12.66 | 2.532 | 2.30 | 0.05328 | 0.05328 |
| LH_Default_PFC_3-LH_Ca | Phenotype | 18.74 | 3.748 | 2.56 | 0.03040 | 0.03040 | 16.65 | 3.331 | 2.27 | 0.05253 | 0.05253 |
| RH_Default_Temp_1-RH_Default_Temp_3 | Group | 15.57 | 7.783 | 3.59 | 0.03078 | 0.03078 | 10.30 | 5.150 | 2.35 | 0.10074 | 0.10074 |
| LH_Default_PFC_4-LH_Pu | Group | 8.00 | 3.999 | 3.52 | 0.03198 | 0.03198 | 1.46 | 0.728 | 0.65 | 0.52386 | 0.52386 |
| RH_Cont_PFCmp_3-RH_Default_PCC_1 | Group | 8.61 | 4.303 | 3.55 | 0.03198 | 0.03198 | 7.65 | 3.825 | 3.05 | 0.05144 | 0.05144 |
| LH_SomMot_6-Cerebellum_Region1 | Group | 7.85 | 3.923 | 3.51 | 0.03269 | 0.03269 | 6.32 | 3.161 | 2.88 | 0.05986 | 0.05986 |
| RH_SalVentAttn_Med_2-RH_Pu | Phenotype | 9.97 | 1.995 | 2.49 | 0.03464 | 0.03464 | 6.46 | 1.291 | 1.65 | 0.15262 | 0.15262 |
| RH_SalVentAttn_Med_2-RH_Pu | Group | 5.64 | 2.821 | 3.45 | 0.03479 | 0.03479 | 2.33 | 1.167 | 1.46 | 0.23711 | 0.23711 |
| LH_Vis_8-LH_Cont_pCun_1 | Group | 4.03 | 2.014 | 3.46 | 0.03521 | 0.03521 | 2.92 | 1.459 | 2.39 | 0.09725 | 0.09725 |
| LH_Default_PFC_3-LH_Ca | Group | 10.17 | 5.086 | 3.40 | 0.03628 | 0.03628 | 8.40 | 4.202 | 2.81 | 0.06406 | 0.06406 |
| RH_SomMot_1-RH_SalVentAttn_TempOccPar_2 | Phenotype | 7.28 | 1.456 | 2.46 | 0.03644 | 0.03644 | 6.69 | 1.338 | 2.28 | 0.05076 | 0.05076 |
| LH_DorsAttn_Post_6-Cerebellum_Region1 | Group | 7.75 | 3.876 | 3.30 | 0.04033 | 0.04033 | 6.12 | 3.062 | 2.73 | 0.06992 | 0.06992 |
| LH_DorsAttn_FEF_1-LH_Limbic_TempPole_2 | Group | 5.25 | 2.625 | 3.29 | 0.04087 | 0.04087 | 3.23 | 1.614 | 1.98 | 0.14279 | 0.14279 |
| RH_Vis_4-RH_Vis_7 | Phenotype | 10.87 | 2.174 | 2.37 | 0.04288 | 0.04288 | 11.05 | 2.209 | 2.24 | 0.05468 | 0.05468 |
| LH_Vis_8-LH_Cont_pCun_1 | Phenotype | 6.86 | 1.371 | 2.39 | 0.04300 | 0.04300 | 5.72 | 1.145 | 1.90 | 0.10134 | 0.10134 |
| LH_SalVentAttn_Med_1-LH_SalVentAttn_Med_3 | Group | 7.52 | 3.759 | 3.23 | 0.04380 | 0.04380 | 4.12 | 2.060 | 1.79 | 0.17279 | 0.17279 |
| LH_SomMot_1-LH_Default_Temp_2 | Group | 6.56 | 3.278 | 3.19 | 0.04487 | 0.04487 | 5.13 | 2.563 | 2.47 | 0.08897 | 0.08897 |
| LH_Cont_Cing_1-LH_Default_PFC_3 | Group | 5.57 | 2.786 | 3.15 | 0.04660 | 0.04660 | 5.07 | 2.534 | 2.72 | 0.07050 | 0.07050 |
| LH_SalVentAttn_Med_3-LH_STH | Group | 6.49 | 3.244 | 3.14 | 0.04676 | 0.04676 | 2.42 | 1.212 | 1.21 | 0.30335 | 0.30335 |
| RH_Vis_5-RH_Limbic_TempPole_1 | Phenotype | 21.83 | 4.366 | 2.31 | 0.04915 | 0.04915 | 19.65 | 3.930 | 2.22 | 0.05842 | 0.05842 |
| LH_DorsAttn_Post_3-LH_Cont_pCun_1 | Phenotype | 8.96 | 1.793 | 2.30 | 0.04920 | 0.04920 | 5.85 | 1.171 | 1.47 | 0.20651 | 0.20651 |
| LH_Default_PFC_3-LH_Pu | Phenotype | 24.62 | 4.923 | 2.28 | 0.05117 | 0.05117 | 25.34 | 5.068 | 2.36 | 0.04534 | 0.04534 |
| RH_Vis_4-RH_Default_PFCv_1 | Group | 5.50 | 2.749 | 3.06 | 0.05142 | 0.05142 | 7.51 | 3.757 | 4.45 | 0.01446 | 0.01446 |
| LH_SomMot_6-Cerebellum_Region5 | Phenotype | 11.70 | 2.341 | 2.27 | 0.05146 | 0.05146 | 11.32 | 2.264 | 2.31 | 0.04860 | 0.04860 |
| RH_DorsAttn_FEF_1-RH_Cont_PFCl_2 | Phenotype | 12.84 | 2.568 | 2.27 | 0.05250 | 0.05250 | 12.97 | 2.595 | 2.38 | 0.04383 | 0.04383 |
| RH_Vis_4-RH_Limbic_TempPole_1 | Phenotype | 11.20 | 2.239 | 2.25 | 0.05302 | 0.05302 | 11.62 | 2.324 | 2.31 | 0.04786 | 0.04786 |
| RH_SalVentAttn_FrOper_1-RH_Default_PFCv_1 | Group | 5.67 | 2.834 | 2.98 | 0.05396 | 0.05396 | 8.00 | 4.001 | 4.11 | 0.01866 | 0.01866 |
| RH_Limbic_TempPole_1-RH_NAC | Phenotype | 9.26 | 1.852 | 2.24 | 0.05443 | 0.05443 | 10.74 | 2.148 | 2.65 | 0.02656 | 0.02656 |
| LH_DorsAttn_Post_3-LH_SalVentAttn_FrOper_1 | Group | 5.88 | 2.941 | 2.98 | 0.05544 | 0.05544 | 6.47 | 3.237 | 3.35 | 0.03918 | 0.03918 |
| LH_Default_PFC_2-LH_Default_PFC_4 | Group | 4.74 | 2.372 | 2.94 | 0.05634 | 0.05634 | 8.05 | 4.025 | 5.11 | 0.00745 | 0.00745 |
| LH_SalVentAttn_Med_1-RH_Default_PCC_2 | Group | 7.80 | 3.899 | 2.91 | 0.05839 | 0.05839 | 9.07 | 4.537 | 3.49 | 0.03370 | 0.03370 |
| RH_SomMot_4-RH_SomMot_6 | Group | 6.40 | 3.200 | 2.88 | 0.06026 | 0.06026 | 7.98 | 3.992 | 3.52 | 0.03331 | 0.03331 |
| LH_Vis_5-LH_Default_PFC_2 | Phenotype | 9.26 | 1.852 | 2.21 | 0.06040 | 0.06040 | 9.94 | 1.988 | 2.40 | 0.04470 | 0.04470 |
| RH_SalVentAttn_Med_2-RH_Default_PCC_2 | Phenotype | 14.15 | 2.831 | 2.18 | 0.06086 | 0.06086 | 15.33 | 3.067 | 2.84 | 0.01898 | 0.01898 |
| RH_Limbic_TempPole_1-RH_Default_PCC_1 | Phenotype | 8.95 | 1.790 | 2.17 | 0.06094 | 0.06094 | 11.77 | 2.354 | 2.91 | 0.01639 | 0.01639 |
| LH_Limbic_OFC_1-LH_Limbic_TempPole_1 | Group | 5.60 | 2.800 | 2.84 | 0.06187 | 0.06187 | 7.79 | 3.895 | 3.96 | 0.02137 | 0.02137 |
| RH_DorsAttn_Post_5-Cerebellum_Region8 | Phenotype | 11.13 | 2.226 | 2.16 | 0.06558 | 0.06558 | 11.41 | 2.281 | 2.48 | 0.03901 | 0.03901 |
| LH_Limbic_TempPole_1-LH_Pulvinar | Phenotype | 7.38 | 1.476 | 2.14 | 0.06615 | 0.06615 | 7.92 | 1.585 | 2.33 | 0.04826 | 0.04826 |
| LH_Vis_1-LH_Limbic_TempPole_1 | Group | 5.04 | 2.519 | 2.75 | 0.06719 | 0.06719 | 7.00 | 3.501 | 3.87 | 0.02325 | 0.02325 |
| LH_Limbic_OFC_1-RH_Limbic_OFC_1 | Group | 5.84 | 2.918 | 2.74 | 0.06827 | 0.06827 | 7.78 | 3.888 | 3.81 | 0.02464 | 0.02464 |
| LH_Limbic_TempPole_1-LH_HTH | Phenotype | 14.42 | 2.884 | 2.10 | 0.06986 | 0.06986 | 17.20 | 3.440 | 2.57 | 0.03031 | 0.03031 |
| LH_DorsAttn_FEF_1-LH_Cont_PFCl_1 | Phenotype | 8.96 | 1.792 | 2.09 | 0.06989 | 0.06989 | 10.30 | 2.060 | 2.39 | 0.04186 | 0.04186 |
| Cerebellum_Region6-Cerebellum_Region7 | Group | 9.92 | 4.962 | 2.72 | 0.06997 | 0.06997 | 11.35 | 5.675 | 3.08 | 0.04990 | 0.04990 |
| RH_Limbic_TempPole_1-RH_Default_PFCm_1 | Group | 5.13 | 2.566 | 2.72 | 0.07070 | 0.07070 | 7.92 | 3.958 | 4.80 | 0.01051 | 0.01051 |
| RH_Vis_4-RH_Default_Temp_2 | Group | 4.12 | 2.058 | 2.69 | 0.07170 | 0.07170 | 4.97 | 2.484 | 3.17 | 0.04581 | 0.04581 |
| RH_DorsAttn_FEF_1-RH_Cont_PFCl_4 | Phenotype | 12.99 | 2.597 | 2.02 | 0.07921 | 0.07921 | 16.49 | 3.297 | 2.69 | 0.02397 | 0.02397 |
| RH_Vis_3-RH_Limbic_TempPole_1 | Phenotype | 9.72 | 1.945 | 2.02 | 0.07946 | 0.07946 | 12.30 | 2.461 | 2.57 | 0.02980 | 0.02980 |
| RH_Cont_PFCl_1-RH_Cont_PFCl_3 | Group | 4.87 | 2.437 | 2.58 | 0.07977 | 0.07977 | 8.35 | 4.173 | 4.71 | 0.01086 | 0.01086 |
| RH_SalVentAttn_Med_2-RH_Default_PCC_2 | Group | 6.79 | 3.396 | 2.56 | 0.08146 | 0.08146 | 8.20 | 4.099 | 3.67 | 0.02867 | 0.02867 |
| LH_Vis_9-RH_Vis_6 | Group | 6.38 | 3.191 | 2.53 | 0.08325 | 0.08325 | 7.76 | 3.879 | 3.16 | 0.04553 | 0.04553 |
| RH_Limbic_TempPole_1-RH_Pu | Phenotype | 18.58 | 3.716 | 1.98 | 0.08550 | 0.08550 | 25.63 | 5.125 | 3.18 | 0.00997 | 0.00997 |
| LH_Limbic_OFC_1-LH_GPe | Phenotype | 12.48 | 2.496 | 2.00 | 0.08584 | 0.08584 | 14.32 | 2.864 | 2.40 | 0.04371 | 0.04371 |
| RH_DorsAttn_Post_5-Cerebellum_Region2 | Phenotype | 12.59 | 2.517 | 1.98 | 0.08624 | 0.08624 | 13.36 | 2.672 | 2.41 | 0.04105 | 0.04105 |
| LH_SalVentAttn_Med_1-LH_SalVentAttn_Med_2 | Phenotype | 10.46 | 2.092 | 1.92 | 0.09626 | 0.09626 | 13.46 | 2.692 | 2.49 | 0.03516 | 0.03516 |
| RH_SomMot_8-Cerebellum_Region3 | Phenotype | 8.15 | 1.629 | 1.91 | 0.09845 | 0.09845 | 9.94 | 1.989 | 2.44 | 0.03993 | 0.03993 |
| LH_SalVentAttn_Med_1-RH_Cont_PFCmp_2 | Group | 6.06 | 3.032 | 2.20 | 0.11490 | 0.11490 | 8.49 | 4.245 | 3.15 | 0.04645 | 0.04645 |
| RH_Vis_6-RH_Default_PCC_1 | Group | 4.55 | 2.273 | 1.97 | 0.14421 | 0.14421 | 7.07 | 3.536 | 3.10 | 0.04968 | 0.04968 |
| Cerebellum_Region2-Cerebellum_Region7 | Group | 5.62 | 2.808 | 1.76 | 0.17569 | 0.17569 | 10.65 | 5.327 | 3.77 | 0.02576 | 0.02576 |
| LH_DorsAttn_FEF_1-LH_Default_PFC_2 | Group | 3.28 | 1.641 | 1.66 | 0.19444 | 0.19444 | 6.80 | 3.399 | 3.68 | 0.02883 | 0.02883 |
| RH_Limbic_TempPole_1-RH_Pu | Group | 6.31 | 3.154 | 1.64 | 0.19855 | 0.19855 | 13.15 | 6.577 | 3.92 | 0.02240 | 0.02240 |
| RH_Vis_4-RH_Pu | Group | 2.86 | 1.429 | 1.58 | 0.21232 | 0.21232 | 6.37 | 3.186 | 3.73 | 0.02809 | 0.02809 |
| RH_Default_PFCm_1-RH_HTH | Group | 6.11 | 3.054 | 1.44 | 0.24351 | 0.24351 | 10.42 | 5.210 | 3.13 | 0.04879 | 0.04879 |
| RH_Default_PFCv_2-RH_Pu | Group | 1.86 | 0.932 | 1.00 | 0.36984 | 0.36984 | 5.42 | 2.712 | 3.15 | 0.04727 | 0.04727 |

## Supplementary Table 6. Group differences in persistent homology for the n=139 subset analysis, assessed by Kolmogorov-Smirnov difference group mean Betti-0 (b0) and Betti-1 (b1) curves. Statistically significant results (alpha = 0.05) are marked with an asterisk. Graph type: GFA = graphs based on untransformed GFA values; W-score: graphs based on w-scores normalized to a 0--1 range based on the global minimum and maximum in the dataset.

| metric | analysis |  | graph_type | Control vs. aAD | Control vs. naAD | aAD vs. naAD |
| --- | --- | --- | --- | --- | --- | --- |
| b0 | full |  | gfa | 6.99 | 8.75 | 2.59 |
|  |  |  | W-score | 16.10 | **23.7*** | 10.00 |
|  | no imputation |  | gfa | 5.75 | 7.64 | 2.54 |
|  |  |  | W-score | 15.50 | **23.8*** | 11.20 |
|  | all biomarker-supported |  | gfa | 9.21 | 8.93 | 1.97 |
|  |  |  | W-score | 16.70 | **23.3*** | 8.00 |
| b1 | full |  | gfa | 20.50 | 21.2 | 8.20 |
|  |  |  | W-score | 11.70 | 17.4 | 8.19 |
|  | no imputation |  | gfa | 17.00 | 17.1 | 8.97 |
|  |  |  | W-score | 8.59 | 15.9 | 10.10 |
|  | all biomarker-supported |  | gfa | 24.50 | 21.8 | 6.86 |
|  |  |  | W-score | 11.90 | 16.5 | 6.21 |

## Supplementary Table 7. Comparison of associations of MMSE and disease duration and Betti metrics in full (n=153) vs. subset (n=139) analyses.

| Outcome | graph_type | betti | estimate | std.error | statistic | p.value | estimate_n139 | std.error_n139 | statistic_n139 | p.value_n139 |
| --- | --- | --- | --- | --- | --- | --- | --- | --- | --- | --- |
| MMSE | GFA | B-0 (# components) | -0.136 | 0.037 | -3.717 | 0.0003 | -0.144 | 0.035 | -4.088 | 0.0001 |
| MMSE | GFA | B-1 (# loops) | 0.043 | 0.014 | 3.195 | 0.0017 | 0.048 | 0.013 | 3.705 | 0.0003 |
| MMSE | GFA | B-1/B-0 | 0.718 | 0.217 | 3.304 | 0.0012 | 0.715 | 0.209 | 3.425 | 0.0008 |
| MMSE | W-score | B-0 (# components) | -0.049 | 0.015 | -3.171 | 0.0018 | -0.055 | 0.015 | -3.610 | 0.0004 |
| MMSE | W-score | B-1 (# loops) | 0.018 | 0.009 | 1.986 | 0.0489 | 0.021 | 0.009 | 2.291 | 0.0235 |
| MMSE | W-score | B-1/B-0 | 0.760 | 0.284 | 2.672 | 0.0084 | 0.811 | 0.275 | 2.943 | 0.0038 |
| Duration | GFA | B-0 (# components) | 0.045 | 0.019 | 2.345 | 0.0209 | 0.049 | 0.018 | 2.631 | 0.0100 |
| Duration | GFA | B-1 (# loops) | -0.015 | 0.007 | -2.090 | 0.0391 | -0.017 | 0.007 | -2.529 | 0.0132 |
| Duration | GFA | B-1/B-0 | -0.243 | 0.141 | -1.731 | 0.0864 | -0.272 | 0.138 | -1.970 | 0.0520 |
| Duration | W-score | B-0 (# components) | 0.016 | 0.007 | 2.290 | 0.0241 | 0.013 | 0.007 | 1.857 | 0.0665 |
| Duration | W-score | B-1 (# loops) | -0.011 | 0.004 | -2.773 | 0.0066 | -0.008 | 0.004 | -2.170 | 0.0326 |
| Duration | W-score | B-1/B-0 | -0.405 | 0.155 | -2.614 | 0.0103 | -0.340 | 0.153 | -2.228 | 0.0284 |
